# Optimal treatment strategies for unresectable stage III *EGFR*-mutated non-small cell lung cancer: a systematic review and Bayesian network meta-analysis

**DOI:** 10.3389/fonc.2026.1852617

**Published:** 2026-06-26

**Authors:** Yang Yang, Jinhan Sun, Ting Luo, Xinfu Liu, Yanyang Wang

**Affiliations:** 1 Department of Oncology, Shaoyang Central Hospital, Shaoyang, Hunan, China; 2 First Clinical Medical College, Ningxia Medical University, Yinchuan, Ningxia, China; 3 Department of Respiratory Medicine,Shaoyang Central Hospital, Shaoyang, Hunan, China; 4 Department of Radiation Oncology, General Hospital of Ningxia Medical University, Yinchuan, Ningxia, China

**Keywords:** chemoradiotherapy, EGFR mutations, EGFR-TKIs, network meta-analysis, unresectable stage III NSCLC

## Abstract

**Background:**

The PACIFIC regimen (consolidation durvalumab following chemoradiotherapy) is the standard of care for unresectable stage III non-small cell lung cancer (NSCLC). With the publication of data from the phase III LAURA trial and the emergence of real-world evidence regarding sequential toxicity, concurrent chemoradiotherapy followed by sequential targeted therapy with EGFR tyrosine kinase inhibitors (TKIs) is recommended for patients with EGFR mutations. However, the optimal combination regimen remains to be determined.

**Methods:**

We systematically searched the PubMed, Embase, Cochrane Library, and Web of Science databases to identify randomized controlled trials (RCTs) and high-quality retrospective studies comparing various therapeutic strategies for unresectable stage III EGFR-mutated NSCLC. The primary endpoints were progression-free survival (PFS) and overall survival (OS), while secondary endpoints included the objective response rate (ORR) and safety profiles. A network meta-analysis (NMA) was performed using a Bayesian random-effects model. Hazard ratios (HRs), odds ratios (ORs), and their corresponding 95% credible intervals (CrIs) were calculated.

**Results:**

A total of 12 studies involving 1,529 patients were analyzed to compare six therapeutic strategies: consolidation durvalumab following chemoradiotherapy (CRT+Durva), CRT alone, consolidation EGFR-TKIs after CRT (CRT+EGFR-TKI), EGFR-TKI monotherapy, EGFR-TKI in combination with chemotherapy (EGFR-TKI+Chemo), and EGFR-TKI integrated with radiotherapy (EGFR-TKI+RT) via induction, concurrent, or consolidation sequencing. NMA revealed that CRT+EGFR-TKI was the only strategy to demonstrate a statistically significant improvement in OS compared to CRT alone (HR = 0.63, 95% CrI: 0.41–0.94), while also achieving the highest ORR. EGFR-TKI+RT (chemotherapy-free regimen) ranked first for PFS (HR = 0.14, 95% CrI: 0.06–0.33) and exhibited a favorable safety profile, associated with the lowest risk of severe radiation pneumonitis (RP). Notably, CRT+Durva failed to yield a survival benefit (PFS: HR = 0.75; OS: HR = 0.82) and was characterized by higher toxicity. An RCT-only sensitivity analysis demonstrated consistent PFS benefits and a comparable OS trend (HR = 0.68, 95% CrI: 0.33–1.4), validating the integration of real-world data to maintain adequate statistical power.

**Conclusions:**

For unresectable stage III EGFR-mutated NSCLC, CRT+EGFR-TKI represents the optimal strategy for extending OS. Conversely, the EGFR-TKI+RT (chemotherapy-free regimen) approach provides a superior balance between prolonged PFS and clinical tolerability.

**Systematic review registration:**

https://www.crd.york.ac.uk/prospero/, identifier CRD420261285935.

## 1 Introduction

Lung cancer remains the leading cause of cancer-related mortality worldwide (1), with approximately 30% of patients with non-small cell lung cancer (NSCLC) presenting with locally advanced disease (Stage III) at initial diagnosis (2). For patients with unresectable disease, definitive concurrent chemoradiotherapy (CRT) followed by sequential consolidation with durvalumab has been established as the global standard of care, predicated on the landmark PACIFIC trial (3). However, this therapeutic paradigm faces significant challenges in the subgroup of patients harboring epidermal growth factor receptor (EGFR) mutations (4). *Post-hoc* analyses and multiple real-world studies have consistently demonstrated that this population derives marginal benefit from immunotherapy (5). This limited efficacy is primarily attributed to the characteristic “cold” tumor microenvironment often exhibited by EGFR-mutated tumors, which is marked by low PD-L1 expression and sparse infiltration of tumor-infiltrating lymphocytes (TILs) (6).

The therapeutic landscape is undergoing a rapid evolution. Building upon recent data from the phase III LAURA and POLESTAR trials, the management of unresectable stage III EGFR-mutated NSCLC has experienced a fundamental paradigm shift between 2024 and 2026, pivoting from “immunotherapy consolidation” toward “targeted consolidation” (7–9). Concurrently, an increasing body of evidence from Asian populations suggests that a “chemotherapy-free” model, which integrates EGFR-TKIs directly with radiotherapy (EGFR-TKI+RT), may offer comparable efficacy while significantly mitigating toxicity (10, 11).

Despite these advancements, clinical decision-making is currently hindered by several critical controversies. First, it remains inconclusive whether integrating chemotherapy (CRT+EGFR-TKI) effectively translates progression-free survival (PFS) gains into a definitive overall survival (OS) advantage, or merely exacerbates toxicity (12, 13). In the era of third-generation TKIs, the feasibility of “chemotherapy-free” models and the determination of optimal TKI-based integration strategies remain active areas of investigation (14). Second, emerging safety signals indicate that potential synergistic toxicities—particularly radiation pneumonitis (RP)—remain a significant concern when thoracic radiotherapy is combined with TKIs or immune checkpoint inhibitors (ICIs) (15, 16). Furthermore, the elevated risk of severe interstitial lung disease (ILD) when TKIs are administered following prior ICI therapy presents a critical clinical challenge that necessitates careful consideration in treatment selection (17).

To address these gaps, this study incorporates the most recent randomized controlled trials (RCTs) and high-quality retrospective cohort studies published up to January 2026. Utilizing a Bayesian network meta-analysis (NMA) framework, we systematically evaluated the relative efficacy and safety of six distinct therapeutic strategies to delineate the optimal treatment pathway for patients with unresectable stage III EGFR-mutated NSCLC.

## 2 Methods

This study was conducted in strict accordance with the PRISMA-NMA (Preferred Reporting Items for Systematic Reviews and Meta-Analyses 2020 Statement for Network Meta-Analysis) guidelines (**Supplementary Table 1** in **Supplementary Appendix S1**). To ensure methodological transparency and reproducibility, the study protocol was prospectively registered in the PROSPERO international database of systematic reviews (Registration No.: CRD420261285935).

### 2.1 Search strategy and selection criteria

We systematically searched the PubMed, Embase, Cochrane Library, and Web of Science databases from their inception through January 13, 2026. The search utilized a combination of Medical Subject Headings (MeSH) terms and keywords, including “non-small cell lung cancer,” “stage III,” “unresectable,” “EGFR mutation,” “chemoradiotherapy,” and “tyrosine kinase inhibitor” (**Supplementary Table 2** in **Supplementary Appendix S1**). To supplement the electronic search, we manually screened abstracts from major international oncology congresses (ASCO, ESMO, and WCLC), as well as the reference lists of retrieved eligible articles.

Studies were considered eligible if they met the following criteria: (1) involved patients with histologically confirmed unresectable stage III NSCLC harboring sensitizing EGFR mutations; (2) designed as RCTs, prospective non-randomized clinical trials, or high-quality retrospective cohort studies comparing two or more therapeutic regimens; (3) reported at least one of the survival or safety metrics (OS, PFS, ORR, or TRAEs). Exclusion criteria were: (1) studies involving patients who received neoadjuvant therapy followed by surgery; (2) studies that included both stage III and stage IV patients where subgroup data for stage III could not be extracted independently; (3) studies lacking primary outcomes or the necessary statistical data to calculate HRs and 95% CIs; (4) overlapping patient cohorts, in which case only the most recent or comprehensive report was included.

### 2.2 Data extraction and quality assessment

Two investigators independently performed literature screening and data extraction. Extracted data included basic study characteristics, patient demographics, details of therapeutic interventions, and clinical outcome data (HRs, ORs, corresponding 95% CIs/CrIs, and event counts). Discrepancies were resolved through consensus or by consultation with a third senior investigator. The risk of bias in the included RCTs was evaluated using the Cochrane Risk of Bias tool 2.0 (RoB 2.0). For non-randomized interventional and retrospective studies, quality assessment was performed using the Newcastle-Ottawa Scale (NOS), with a score of ≥7 considered high-quality.

### 2.3 Statistical analysis

Bayesian NMA was performed using the *gemtc* package in R (version 4.5.2), interfacing with JAGS software, utilizing random-effects models. We implemented four Markov Chain Monte Carlo (MCMC) chains, each consisting of 60,000 iterations, with the initial 10,000 iterations discarded as a burn-in period. Model convergence was assessed using the Brooks-Gelman-Rubin diagnostic (Potential Scale Reduction Factor, PSRF); convergence was determined when the PSRF approached 1.00 and trace plots demonstrated sufficient mixing.

To evaluate the robustness of the NMA results, multi-dimensional sensitivity analyses were performed. Local inconsistency was assessed via the node-splitting method; if significant inconsistency (*P* < 0.05) or moderate-to-high heterogeneity (*I^2^* > 50%) was detected, a “leave-one-out” analysis was employed to identify potential outlier studies. The appropriateness of model selection was verified using the Deviance Information Criterion (DIC). For survival data (PFS, OS), the effect size was expressed as the HR with its 95% CrI, while dichotomous variables (ORR, TRAEs, RP) were expressed as the OR with its 95% CrI. Finally, rank probability plots and Surface Under the Cumulative Ranking (SUCRA) curves were utilized to determine the optimal treatment regimen.

Furthermore, to evaluate the potential impact of study design heterogeneity on the pooled effect sizes—particularly regarding OS and PFS outcomes—we performed a pre-specified sensitivity analysis by restricting the network framework exclusively to RCTs and excluding all retrospective cohort studies.

## 3 Results

### 3.1 Study selection and characteristics

**Figure 1:**
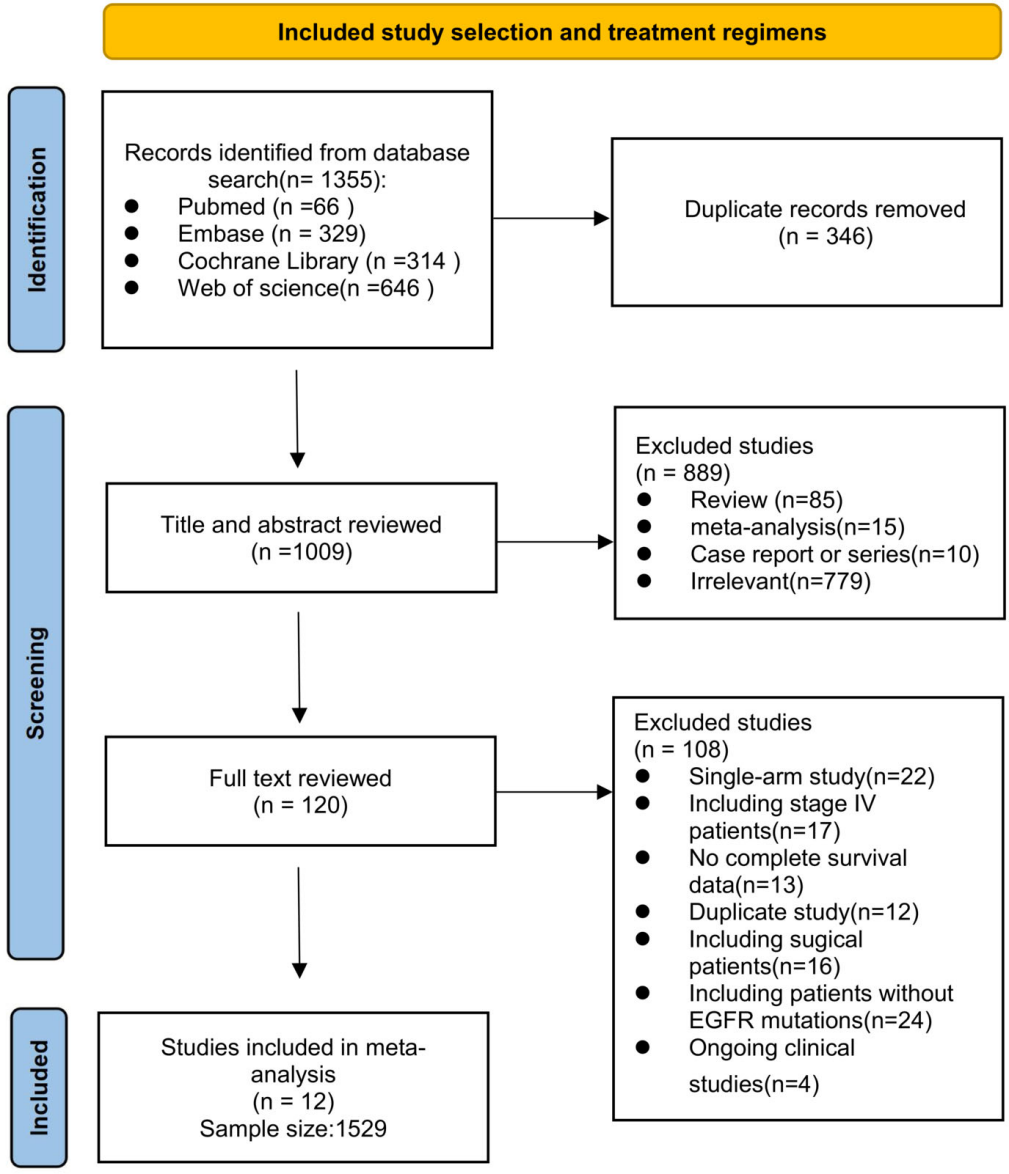
Flow diagram of study selection.

A total of 1,355 relevant records were initially identified. Following the removal of duplicates and eligibility assessments, 12 studies [comprising 5 RCTs (18–22) and 7 retrospective studies (10, 23–28)] involving a cumulative total of 1,529 patients were ultimately included. The detailed study selection process is illustrated in the PRISMA flow diagram (**Figure 1**). The included studies covered six primary therapeutic strategies: CRT+Durva, CRT alone, CRT+EGFR-TKI, EGFR-TKI monotherapy, EGFR-TKI+Chemo, and EGFR-TKI+RT (including induction, concurrent, or consolidation settings in chemotherapy-free regimens). All populations consisted of patients with unresectable stage III EGFR-mutated NSCLC. Detailed baseline characteristics of the included studies and participants are summarized in **Table 1**.

**Table 1:** Basic characteristics of the enrolled studies and participants.

Study	Year	Type	Experiment	Control	Sample size (Experiment/control)	Median follow-up duration (months)	Median ages (years, experiment/control)	Male/female	Outcome
POLESTAR	2024	RCT	CRT+EGFR-TKI	CRT	92 vs 50	16.6 vs 14.9	59 vs 58	61 vs 81	PFS,ORR,AE
NEJ063	2025	Retrospective study	CRT+Durva	CRT	56 vs 56	NA	69 vs 68	40 vs 72	PFS
Nassar et al.	2022	Retrospective study	CRT+Durva, CRT+EGFR-TKI	CRT	56,33 vs 47	48,22 vs 64	67,65 vs 64	48 vs 88	PFS,OS,AE
LAURA	2024	RCT	CRT+EGFR-TKI	CRT	143 vs 73	24 vs 8.3	62 vs 64	84 vs 132	PFS,OS,ORR,AE
Liang et al.	2024	Retrospective study	EGFR-TKI	TKI+chemo	55 vs 23	19	NA	37 vs 41	PFS,OS
ADVANCE	2024	RCT	EGFR-TKI+RT	CRT	24 vs 19	25.5	NA	NA	PFS,OS,AE
PACIFICl	2023	RCT	CRT+Durva	CRT	24 vs 11	42.7	65 vs 69	21 vs 14	PFS,OS,ORR,AE
REFRACT	2023	Retrospective study	CRT+EGFR-TKI,EGFR-TKI	CRT	105,231 vs 104	35.9	56,63 vs 58	175 vs 265	PFS,OS
Wang et al.	2022	Retrospective study	EGFR-TKI	CRT	40 vs 10	NA	71 vs 64	23 vs 27	PFS,OS
RECEL	2021	RCT	EGFR-TKI+RT	CRT	20 vs 21	NA	59.5 vs 59	17 vs 24	PFS,OS,ORR,AE
Aredo et al.	2021	Retrospective study	CRT+Durva,CRT+EGFR-TKI	CRT	13,8 vs 16	21.8	NA	8 vs 29	PFS,AE
Hsia et al.	2018	Retrospective study	EGFR-TKI	CRT	177 vs 22	23	70.2 vs 60.1	77 vs 122	OS

### 3.2 Risk of bias assessment

Risk of bias for the 5 RCTs was summarized using RoB 2.0. The RECEL trial (21) was classified as “high risk,” mainly driven by potential biases in missing outcome data (D3) and outcome measurement (D4) inherent to its open-label phase II design. All 7 retrospective cohort studies achieved an NOS score of ≥7, indicating high quality. Details of the bias assessment are provided in **Supplementary Figure 1** in **Supplementary Appendix S1**.

### 3.3 Network structure and model convergence

**Figure 2:**
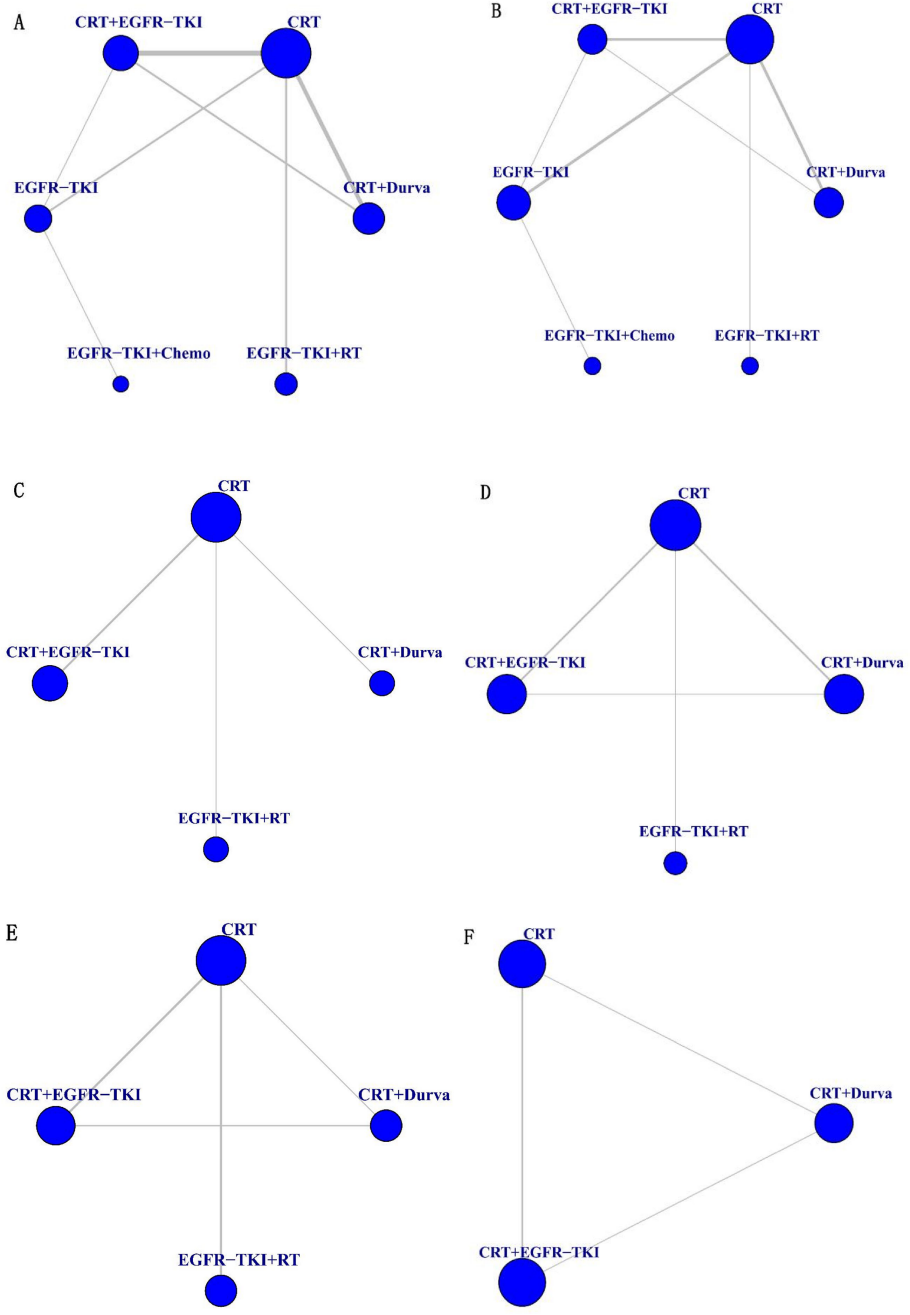
Comparative network plots for unresectable stage III EGFR-mutated NSCLC. A Bayesian framework is used for comparison. **(A)** PFS. **(B)** OS. **(C)** ORR. **(D)** All-grade TRAEs. **(E)** TRAEs of grade greater than or equal to 3. **(F)** RP. Each circle represents a treatment. CRT, Chemoradiotherapy; EGFR-TKI, Epidermal growth factor receptor-Tyrosine kinase inhibitor; EGFR-TKI+Chemo, EGFR-TKI in combination with chemotherapy; EGFR-TKI+RT, EGFR-TKI integrated with radiotherapy; CRT+Durva, durvalumab following chemoradiotherapy; PFS, Progression-free survival; OS, Overall survival; ORR, Objective response rate; TRAEs, Treatment-related adverse events; RP, Radiation pneumonitis.

The network plot (**Figure 2**) illustrates a robust and interconnected structure, with the connection between CRT+EGFR-TKI and CRT being the most prominent. Convergence diagnostics revealed that the PSRF for all assessed metrics (PFS, OS, ORR, and TRAEs) reached 1.00, demonstrating optimal convergence of the MCMC chains. The minimal disparity in the DIC between consistency and inconsistency models confirmed that the consistency model provided an adequate fit.

### 3.4 Efficacy analysis

**Figure 3:**
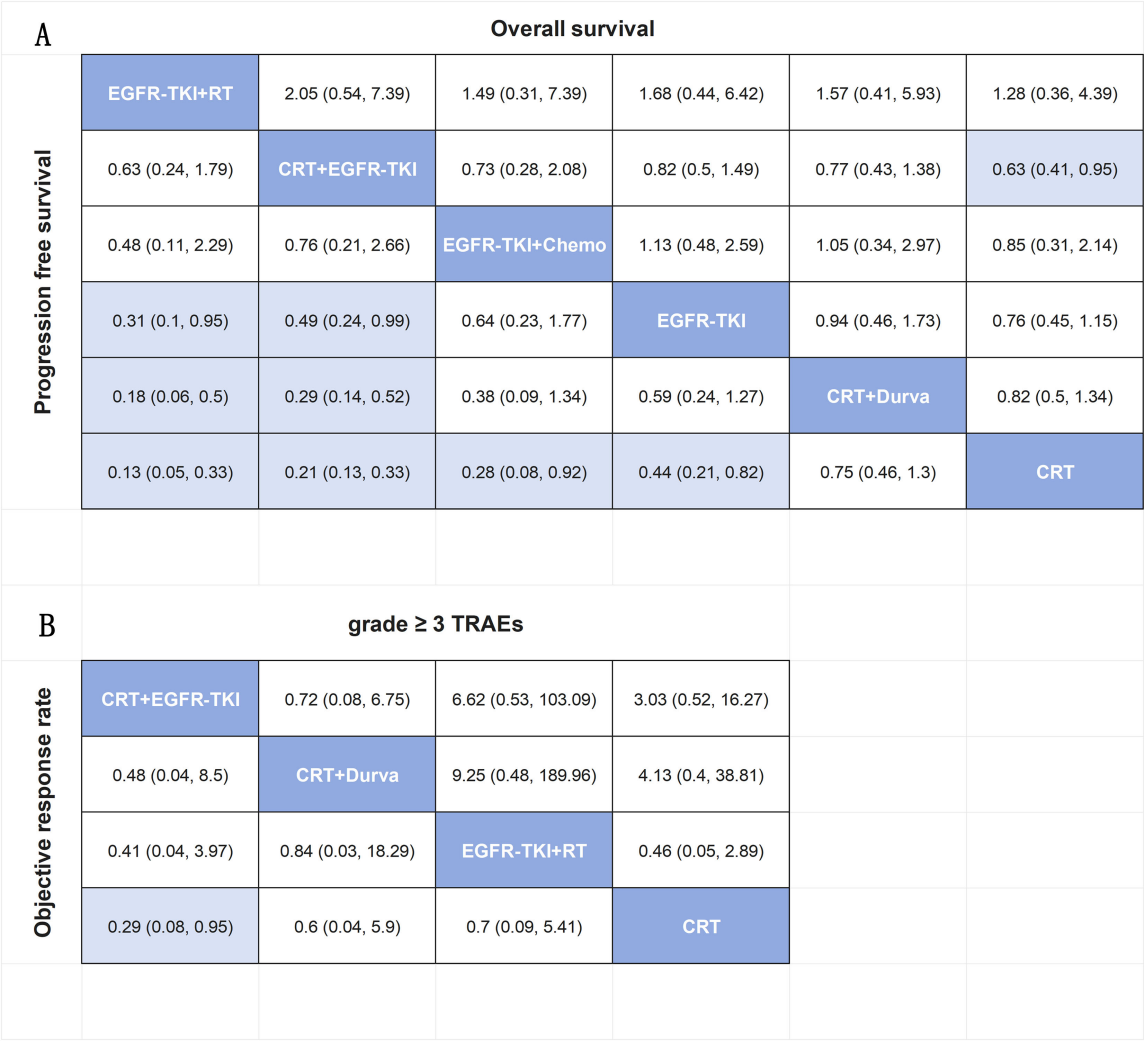
Pooled estimates of the network meta-analysis. **(A)** Pooled hazard ratios (95% credible intervals) for OS (upper triangle) and PFS (lower triangle). **(B)** Pooled odds ratios (95% credible intervals) for grade 3 or higher treatment related adverse events (≥3 TRAEs) (upper triangle) and objective response rate (ORR) (lower triangle). PFS, Progression-free survival; OS, Overall survival; ORR, Objective response rate; TRAEs, Treatment-related adverse events; RP, Radiation pneumonitis.

The overall efficacy and safety profiles of the diverse regimens compared to the CRT reference arm are presented via forest plots in **Supplementary Figure 2** in **Supplementary Appendix S1**. Furthermore, the comprehensive league table detailing pairwise comparisons of all strategies for PFS, OS, ORR, and severe TRAEs is provided in **Figure 3**.

**Figure 4:**
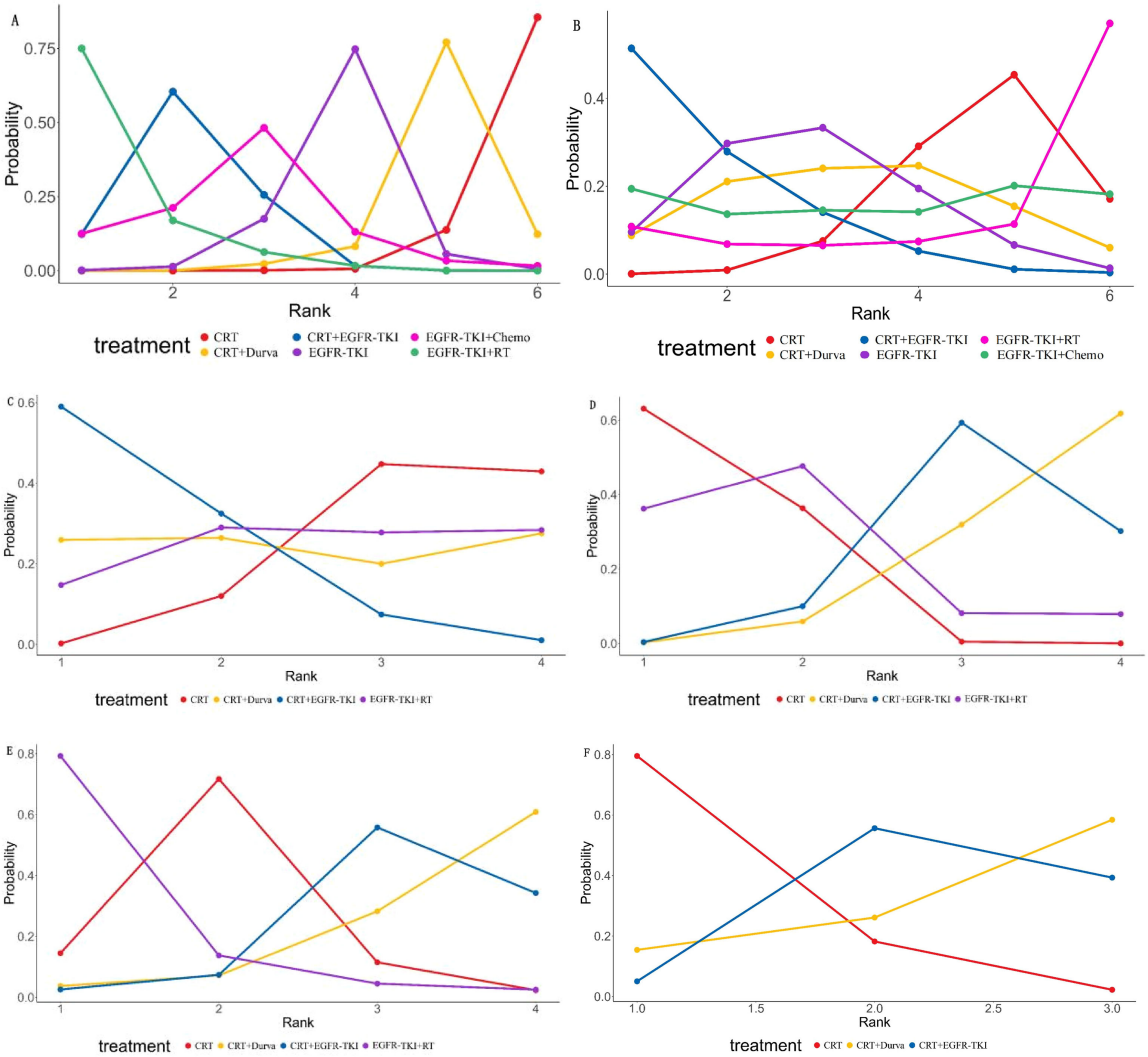
Bayesian ranking profiles of comparable treatments for unresectable stage III EGFR-mutated NSCLC in terms of efficacy and safety. The line graph shows the probability of ranking from first to last for each treatment in terms of PFS, OS, ORR, safety. **(A)** PFS. **(B)** OS. **(C)** ORR. **(D)** Safety assessed according to any-grade TRAEs. **(E)** Safety assessed according to grade greater than or equal to 3 TRAEs. **(F)** RP. PFS, Progression-free survival; OS, Overall survival; ORR, Objective response rate; TRAEs, Treatment related adverse events; RP, Radiation pneumonitis; CRT, Chemoradiotherapy; Durva, Durvalumab; EGFR-TKI, Epidermal growth factor receptor-tyrosine kinase inhibitor; RT, radiotherapy; Chemo, chemotherapy.

Progression-Free Survival (PFS): With CRT as the reference, all EGFR-TKI-containing combination regimens demonstrated significant PFS benefits (**Supplementary Figure 2A**). The EGFR-TKI+RT regimen exhibited the most substantial benefit (HR = 0.13, 95% CrI: 0.05–0.32), with an approximately 75% probability of ranking first in the SUCRA analysis. This was followed by the CRT + EGFR-TKI regimen (HR = 0.22, 95% CrI: 0.14–0.33), which ranked second with a SUCRA value of 60.5% (**Figure 4A**). Notably, the difference between CRT+Durva and CRT was not statistically significant (HR = 0.75, 95% CrI: 0.46–1.30), confirming that immune consolidation failed to provide a PFS benefit.

Overall Survival (OS): CRT+EGFR-TKI was the only regimen to demonstrate a statistically significant benefit compared to CRT (HR = 0.63, 95% CrI: 0.40–0.95) (**Supplementary Figure 2B** in **Supplementary Appendix S1**) and was identified as the optimal strategy for improving OS. Although its SUCRA value was relatively modest at 51.4% (**Figure 4B**), this was primarily due to the wide credible intervals of other comparative regimens limiting overall ranking separation. Other regimens such as EGFR-TKI+RT (HR = 1.30) and CRT+Durva (HR = 0.81) showed no significant long-term survival advantage over CRT.

Objective Response Rate (ORR): Compared to CRT, CRT+EGFR-TKI achieved an OR of 3.5 (95% CrI: 1.1–12) (**Supplementary Figure 2C** in **Supplementary Appendix S1**). According to SUCRA score ranking analysis, CRT+EGFR-TKI (SUCRA, 59%) may be the best choice for ORR benefit (**Figure 4C**). No significant differences were observed for EGFR-TKI+RT (OR = 1.40) or CRT+Durva (OR = 1.70).

Sensitivity Analysis for Study Design: In the sensitivity analysis restricted exclusively to the five RCTs, the network meta-analysis yielded efficacy estimates that were highly consistent with the primary analysis. For PFS, both EGFR-TKI+RT (HR = 0.14, 95% CrI: 0.037–0.45) and CRT+EGFR-TKI (HR = 0.18, 95% CrI: 0.057–0.56) sustained a pronounced and statistically significant advantage over CRT alone. Regarding OS, the point estimate for the CRT+EGFR-TKI regimen (HR = 0.68, 95% CrI: 0.33–1.40) remained closely aligned with that of the primary analysis (HR = 0.63), indicating a consistent trend toward survival benefit. Although this OS advantage lost statistical significance in the restricted network—likely due to the substantially reduced sample size and immature OS events when retrospective cohorts were excluded—the unshifted effect size indicates that our primary estimates were robust. The detailed forest plots and ranking profiles for this RCT-only analysis are provided in **Supplementary Figure 3**.

### 3.5 Safety analysis

Compared with CRT, both CRT+Durva (OR = 13) and CRT+EGFR-TKI (OR = 8.90) were associated with a significantly higher risk of all-grade TRAEs (**Supplementary Figure 2D**) (**Figure 4D**). Regarding Grade ≥3 TRAEs, no regimens reached statistical significance compared to CRT (**Supplementary Figure 2E**); however, distinct trends emerged. Consolidation with either immunotherapy (OR = 4.20) or targeted therapy (OR = 3.0) following chemoradiotherapy markedly increased risk point estimates for severe toxicity (**Figure 4E**). Conversely, EGFR-TKI+RT exhibited a favorable safety profile with a lower risk estimate for severe AEs than CRT (OR = 0.46; 95% CrI: 0.05–2.80). In the independent NMA for RP, CRT+Durva carried the highest risk (OR = 1.80; 95% CrI: 0.35–14.50), while the OR for CR+EGFR-TKI was 1.6 (95% CrI, 0.85–2.90) (**Supplementary Figure 2F**). These data suggest that while adding Durvalumab or EGFR-TKI to CRT may numerically increase the risk of respiratory toxicity (**Figure 4F**), it does not demonstrate a statistically significant elevation in RP risk compared to standard CRT. While the relative ORs indicate elevated risks of all-grade TRAEs for both CRT+Durva and CRT+EGFR-TKI, this apparent paradox compared to the non-significant RP risk is driven by the composition of these adverse events. The significantly elevated overall TRAEs in the targeted and immune consolidation arms are predominantly composed of non-pulmonary, low-grade toxicities specific to the systemic agents (e.g., skin rash, diarrhea, and paronychia for EGFR-TKIs; hematological toxicities from chemotherapy). When isolating the critical pulmonary-specific toxicity, the absolute event rates of severe RP were numerically higher but lacked significant relative differences due to wide credible intervals in our network estimation.

### 3.6 Comprehensive ranking profiles

**Figure 5:**
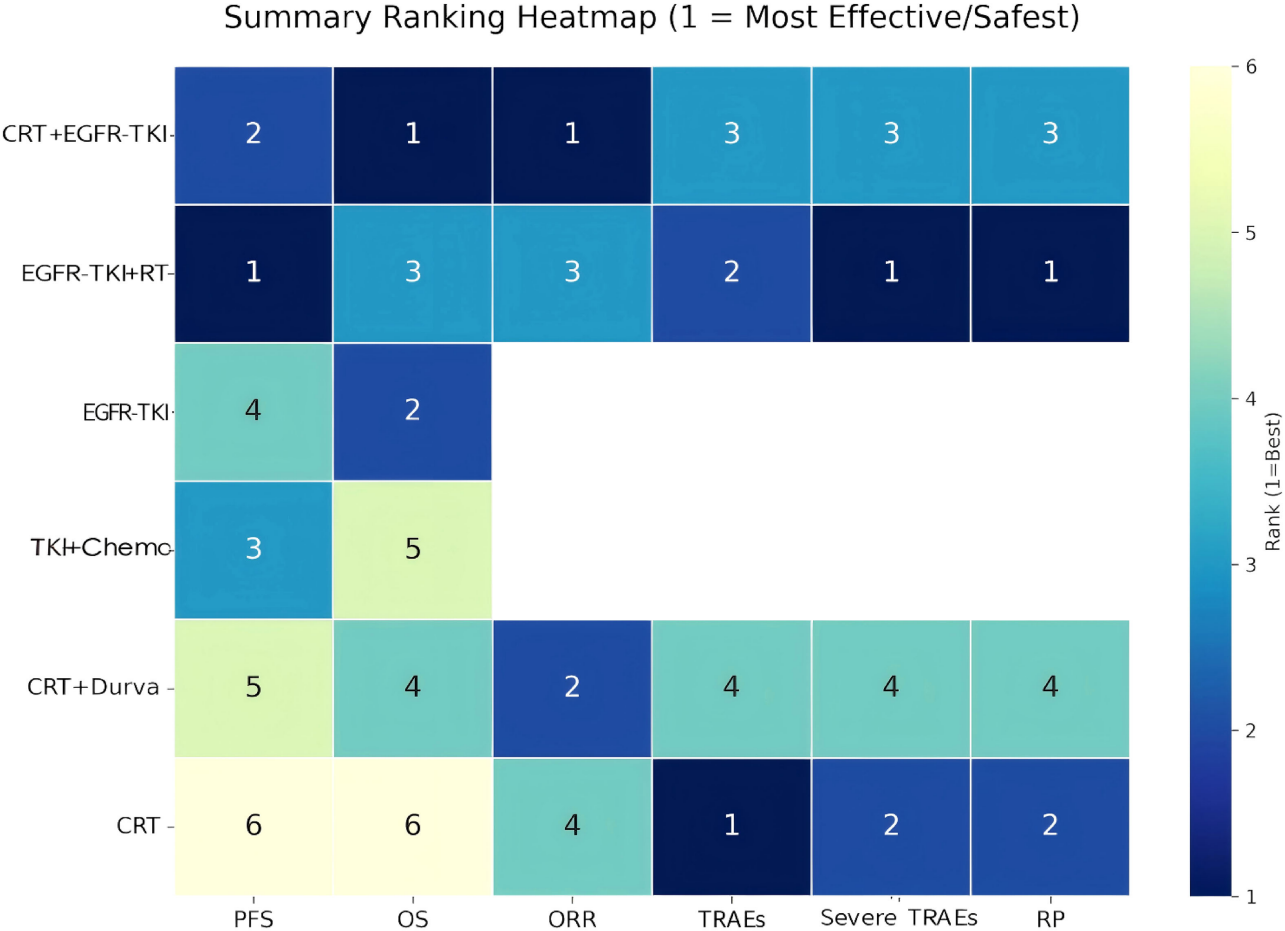
Heatmap summarizing the clinical rankings of treatment strategies across multiple endpoints. Each cell contains a numerical rank (1 = most effective/safest; 6 = least effective/safest), with the color gradient ranging from dark blue (top rank) to light yellow (bottom rank). PFS, progression-free survival; OS, overall survival; ORR, objective response rate; TRAEs, treatment related adverse event; Severe TRAEs, Grade ≥ 3 treatment related adverse events; RP, radiation pneumonitis; CRT, Chemoradiotherapy; Durva, Durvalumab; EGFR-TKI, Epidermal growth factor receptor-tyrosine kinase inhibitor; RT, radiotherapy; Chemo, chemotherapy.

To synthesize the multidimensional outcomes, a summary ranking heatmap was generated based on the Surface Under the Cumulative Ranking (SUCRA) probabilities (**Figure 5**).

### 3.7 Publication bias

Visual inspection of the funnel plots for all major clinical endpoints (PFS, OS, ORR, and safety outcomes) demonstrated visually symmetrical distributions of the included studies. This assessment was statistically confirmed by Egger’s tests across all evaluated endpoints: PFS (intercept = -1.568, *P* = 0.3502), OS (intercept = -0.390, *P* = 0.6589), ORR (intercept = -1.314, *P* = 0.4231), all-grade TRAEs (intercept = 0.543, *P* = 0.7302), grade ≥ 3 TRAEs (intercept = -2.838, *P* = 0.1514), and RP (intercept = -1.127, *P* = 0.3158). These findings consistently indicate no significant evidence of publication bias or small-study effects across the network. The comprehensive funnel plots for all endpoints are provided in **Supplementary Figure 4**.

### 3.8 Network inconsistency and sensitivity analyses

To formally evaluate local inconsistency between direct and indirect comparisons, node-splitting analyses were performed. No significant inconsistency was observed for PFS, OS, and severe toxicity outcomes (Grade ≥ 3 TRAEs and RP) (all P > 0.05; detailed in **Supplementary Table 3**). However, initial assessments revealed local inconsistency and moderate heterogeneity specifically for any-grade TRAEs (*I^2^* = 62.51%). Through a pre-specified ‘leave-one-out’ sensitivity analysis, we determined this inconsistency originated from a single retrospective study featuring a relatively small sample size and an extreme outlier effect size. Upon its exclusion, the model consistency improved significantly without altering the overall safety ranking trends (**Supplementary Table 4**).

## 4 Discussion

This NMA provides a comprehensive comparison of therapeutic strategies for unresectable stage III EGFR-mutated NSCLC. Following the confirmation of robust model convergence, our findings fundamentally reshape the treatment hierarchy for this population.

We identified consolidation EGFR-TKI following CRT as the optimal strategy for long-term survival, being the only intervention to demonstrate a statistically significant benefit in OS (HR = 0.63) and achieving the highest ORR (HR = 3.50). These results align with recent data from the LAURA trial, which demonstrated that osimertinib consolidation reduced the risk of disease progression or death by 84% (29). Furthermore, real-world evidence supports the efficacy of third-generation TKIs combined with radiotherapy in controlling intracranial metastases (10). Notably, the integration of RT with EGFR-TKI in chemotherapy-free paradigms proved most effective for local control, ranking first in PFS (HR = 0.13). Although the OS benefit did not reach statistical significance, studies such as RECEL suggest that removing chemotherapy does not compromise long-term survival in select patients (30).

While CRT+EGFR-TKI yielded definitive survival advantages, it was associated with the highest risk of all-grade TRAEs (OR = 8.90). However, it is crucial to recognize that this elevated overall risk is largely driven by the additive effect of non-pulmonary, predictable, and manageable TKI-specific adverse events (such as dermatologic and gastrointestinal toxicities) alongside chemotherapy-induced hematologic toxicities. Crucially, its relative risk of the most feared synergistic complication—severe radiation pneumonitis (RP)—was comparable to CRT alone (OR = 1.60). This demonstrates that the addition of an EGFR-TKI does not significantly compound specific pulmonary toxicity. Consequently, provided proactive management of typical TKI-related side effects is implemented, for patients with a robust performance status (ECOG PS 0–1), CRT+EGFR-TKI should be established as the preferred standard of care. A pivotal finding of this study is that the chemotherapy-free paradigm (EGFR-TKI+RT) achieved the most favorable risk-benefit ratio. Despite historical concerns regarding overlapping toxicities, this regimen demonstrated the lowest risk of Grade≥3 TRAEs (OR = 0.46). The utilization of third-generation TKIs likely contributes to this superior safety profile. These results suggest that for elderly or frail patients, EGFR-TKI+RT represents a highly effective and viable alternative.

Our data indicate that CRT+Durvalumab yields no significant benefit in either PFS (HR = 0.75) or OS (HR = 0.81). This aligns with findings suggesting that EGFR-mutant tumors harbor an immunosuppressive microenvironment rendered largely refractory to PD-L1 inhibition (23). Beyond the lack of efficacy, real-world evidence highlights a severe risk: the ultra-long half-life of durvalumab can lead to prolonged T-cell activation, which dramatically exacerbates TKI-induced interstitial lung disease (ILD) if an EGFR-TKI is introduced sequentially (24). Initiating osimertinib within six months of the last immunotherapy dose has been reported to result in a 17.6% rate of Grade≥3 RP (23). These findings suggest that ICIs may induce a long-term “immune priming” effect on pulmonary tissue, which is subsequently amplified by sequential TKI therapy. Consequently, based on the currently available exploratory evidence, the routine use of the PACIFIC regimen in the EGFR-mutant population may not be the optimal choice. Future prospective head-to-head phase III trials are warranted to definitively confirm these findings.

While our results strongly support TKI-based regimens for common mutations (exon 19 del/L858R), findings require further refinement for rare EGFR mutations (e.g., G719X, exon 20 insertions). Research demonstrates that within this specific subgroup, patients receiving EGFR-TKIs experienced a significantly shorter PFS compared to those receiving standard CRT (5.0 months vs. 11.9 months, *P* = 0.02) (31). Current evidence suggests that standard CRT remains the preferred strategy for patients with rare EGFR mutations until more robust evidence for mutation-specific novel agents becomes available (31, 32).

A primary limitation of this study is the inherent risk of selection bias and unmeasured confounding introduced by combining RCTs with retrospective cohort studies. However, our sensitivity analysis—restricted solely to RCTs—demonstrated that the PFS benefits of targeted regimens remained statistically significant and highly consistent with the primary analysis. Regarding OS, the RCT-only analysis revealed a stable point estimate for CRT+EGFR-TKI (HR = 0.68) compared to the primary analysis (HR = 0.63). Although OS lost statistical significance in the restricted network due to diminished power, this consistent trend justifies incorporating high-quality real-world data to maintain adequate statistical power. Additionally, clinical heterogeneity within the treatment nodes cannot be ignored. First, pooling 1st- and 3rd-generation TKIs may obscure agent-specific advantages, particularly regarding intracranial efficacy. Nevertheless, because contemporary trials predominantly utilizing 3rd-generation TKIs (e.g., LAURA, POLESTAR) carry the most statistical weight, our estimates remain highly reflective of modern practice. Second, our network operates under a broad “EGFR-mutated” umbrella. Because classic mutations (exon 19 deletion/L858R) constitute the overwhelming majority of enrolled patients, our primary rankings are strictly applicable to this subgroup. Extrapolating these TKI-favored strategies to rare mutations (e.g., exon 20 insertions) is clinically inappropriate, standard CRT currently remains their preferred regimen. Finally, the data regarding rare EGFR mutations remain sparse, lacking sufficient statistical power to provide definitive guidance for this highly heterogeneous subgroup. As ongoing prospective trials mature, the publication of mutation-specific survival data will be vital to refine these network estimates and optimize personalized clinical workflows.

## 5 Conclusions

This NMA demonstrates that consolidation EGFR-TKI following CRT is the most effective strategy for improving OS in patients with unresectable stage III EGFR-mutated NSCLC and should be considered the preferred regimen for those with a robust performance status. Furthermore, the chemotherapy-free paradigm (EGFR-TKI+RT) exhibited the most favorable risk-benefit ratio, offering a viable alternative for elderly patients or those intolerant to chemotherapy. Conversely, the use of consolidation durvalumab following CRT should be carefully reconsidered in this specific molecular subgroup due to its limited efficacy and the heightened risk of cumulative toxicities during subsequent treatment lines. Comprehensive molecular profiling prior to the initiation of definitive CRT is paramount to ensure that patients receive targeted consolidation therapy while avoiding ineffective treatments and the potential toxic risks associated with the indiscriminate use of ICIs.

## Data Availability

The original contributions presented in the study are included in the article/**Supplementary Material**. Further inquiries can be directed to the corresponding authors.
