# Supplementary material for: Optimal treatment strategies for unresectable stage III EGFR-mutated non-small cell lung cancer: a systematic review and Bayesian network meta-analysis
Source: Front Oncol. 2026 Jun 26;16:1852617. doi: 10.3389/fonc.2026.1852617 (PMC13349829; doi:10.3389/fonc.2026.1852617)
Supplement: Supplementary file 1 [file DataSheet1.docx]

**Supplement Materials**

Optimal Treatment Strategies for Unresectable Stage III EGFR-Mutated Non-Small Cell Lung Cancer: A Systematic Review and Bayesian Network Meta-Analysis

| Supplement Table 1: PRISMA 2020 checklist. |
| --- |
| Supplement Table 2: Search strategies for PubMed, Embase, Cochrane Library, and Web of Science databases. |
| Supplement Table 3: Node-splitting analyses. |
| Supplement Table 4: Leave-one-out sensitivity analysis. |
| Supplement Figure 1: The summary of the risk of bias assessment. |
| Supplement Figure 2: Forest plot of the network meta-analysis. |
| Supplement Figure 3: Forest plots and ranking profiles for RCT-only analysis. |
| Supplement Figure 4: Publication Bias. |

**Supplement Table 1**


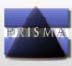
**PRISMA 2020 Checklist**

| **Section and Topic** | **Item #** | **Checklist item** | **Location where item is reported** |
| --- | --- | --- | --- |
| **TITLE** | | |  |
| Title | 1 | Identify the report as a systematic review. | 1 |
| **ABSTRACT** | | |  |
| Abstract | 2 | See the PRISMA 2020 for Abstracts checklist. | 1-2 |
| **INTRODUCTION** | | |  |
| Rationale | 3 | Describe the rationale for the review in the context of existing knowledge. | 3 |
| Objectives | 4 | Provide an explicit statement of the objective(s) or question(s) the review addresses. | 4 |
| **METHODS** | | |  |
| Eligibility criteria | 5 | Specify the inclusion and exclusion criteria for the review and how studies were grouped for the syntheses. | 5-6 |
| Information sources | 6 | Specify all databases, registers, websites, organisations, reference lists and other sources searched or consulted to identify studies. Specify the date when each source was last searched or consulted. | 5 |
| Search strategy | 7 | Present the full search strategies for all databases, registers and websites, including any filters and limits used. | 5 |
| Selection process | 8 | Specify the methods used to decide whether a study met the inclusion criteria of the review, including how many reviewers screened each record and each report retrieved, whether they worked independently, and if applicable, details of automation tools used in the process. | 5-6 |
| Data collection process | 9 | Specify the methods used to collect data from reports, including how many reviewers collected data from each report, whether they worked independently, any processes for obtaining or confirming data from study investigators, and if applicable, details of automation tools used in the process. | 6 |
| Data items | 10a | List and define all outcomes for which data were sought. Specify whether all results that were compatible with each outcome domain in each study were sought (e.g. for all measures, time points, analyses), and if not, the methods used to decide which results to collect. | 6 |
|  | 10b | List and define all other variables for which data were sought (e.g. participant and intervention characteristics, funding sources). Describe any assumptions made about any missing or unclear information. | 6 |
| Study risk of bias assessment | 11 | Specify the methods used to assess risk of bias in the included studies, including details of the tool(s) used, how many reviewers assessed each study and whether they worked independently, and if applicable, details of automation tools used in the process. | 6 |
| Effect measures | 12 | Specify for each outcome the effect measure(s) (e.g. risk ratio, mean difference) used in the synthesis or presentation of results. | 6 |
| Synthesis methods | 13a | Describe the processes used to decide which studies were eligible for each synthesis (e.g. tabulating the study intervention characteristics and comparing against the planned groups for each synthesis (item #5)). | 7 |
|  | 13b | Describe any methods required to prepare the data for presentation or synthesis, such as handling of missing summary statistics, or data conversions. | 7 |
|  | 13c | Describe any methods used to tabulate or visually display results of individual studies and syntheses. | 7 |
|  | 13d | Describe any methods used to synthesize results and provide a rationale for the choice(s). If meta-analysis was performed, describe the model(s), method(s) to identify the presence and extent of statistical heterogeneity, and software package(s) used. | 7 |
|  | 13e | Describe any methods used to explore possible causes of heterogeneity among study results (e.g. subgroup analysis, meta-regression). | 7 |
|  | 13f | Describe any sensitivity analyses conducted to assess robustness of the synthesized results. | 7 |
| Reporting bias assessment | 14 | Describe any methods used to assess risk of bias due to missing results in a synthesis (arising from reporting biases). | 7 |
| Certainty assessment | 15 | Describe any methods used to assess certainty (or confidence) in the body of evidence for an outcome. | 7 |
| **RESULTS** | | |  |
| Study selection | 16a | Describe the results of the search and selection process, from the number of records identified in the search to the number of studies included in the review, ideally using a flow diagram. | 7 |
|  | 16b | Cite studies that might appear to meet the inclusion criteria, but which were excluded, and explain why they were excluded. | 7 |
| Study characteristics | 17 | Cite each included study and present its characteristics. | 8, Table 1 |
| Risk of bias in studies | 18 | Present assessments of risk of bias for each included study. | 8 |
| Results of individual studies | 19 | For all outcomes, present, for each study: (a) summary statistics for each group (where appropriate) and (b) an effect estimate and its precision (e.g. confidence/credible interval), ideally using structured tables or plots. | Table 1,Figure 3, Supplementary Figure 2 |
| Results of syntheses | 20a | For each synthesis, briefly summarise the characteristics and risk of bias among contributing studies. | 5 |
|  | 20b | Present results of all statistical syntheses conducted. If meta-analysis was done, present for each the summary estimate and its precision (e.g. confidence/credible interval) and measures of statistical heterogeneity. If comparing groups, describe the direction of the effect. | 8-9 |
|  | 20c | Present results of all investigations of possible causes of heterogeneity among study results. | 13 |
|  | 20d | Present results of all sensitivity analyses conducted to assess the robustness of the synthesized results. | 13 |
| Reporting biases | 21 | Present assessments of risk of bias due to missing results (arising from reporting biases) for each synthesis assessed. | 10 |
| Certainty of evidence | 22 | Present assessments of certainty (or confidence) in the body of evidence for each outcome assessed. | 13 |
| **DISCUSSION** | | |  |
| Discussion | 23a | Provide a general interpretation of the results in the context of other evidence. | 10-11 |
|  | 23b | Discuss any limitations of the evidence included in the review. | 12 |
|  | 23c | Discuss any limitations of the review processes used. | 12 |
|  | 23d | Discuss implications of the results for practice, policy, and future research. | 13 |
| **OTHER INFORMATION** | | |  |
| Registration and protocol | 24a | Provide registration information for the review, including register name and registration number, or state that the review was not registered. | 5 |
|  | 24b | Indicate where the review protocol can be accessed, or state that a protocol was not prepared. | 5 |
|  | 24c | Describe and explain any amendments to information provided at registration or in the protocol. | No |
| Support | 25 | Describe sources of financial or non-financial support for the review, and the role of the funders or sponsors in the review. | 14 |
| Competing interests | 26 | Declare any competing interests of review authors. | 14 |
| Availability of data, code and other materials | 27 | Report which of the following are publicly available and where they can be found: template data collection forms; data extracted from included studies; data used for all analyses; analytic code; any other materials used in the review. | 14 |

**Supplement Table 2**

Detail search strategies for PubMed, Embase, Cochrane Library, and Web of Science databases.

| Pubmed |
| --- |
| (((((((((((((((Carcinoma, Non-Small-Cell Lung[Title]) OR (Carcinoma, Non Small Cell Lung[Title])) OR (Carcinomas, Non-Small-Cell Lung[Title])) OR (Lung Carcinoma, Non-Small-Cell[Title])) OR (Lung Carcinomas, Non-Small-Cell[Title])) OR (Non-Small-Cell Lung Carcinomas[Title])) OR (Carcinoma, Non-Small Cell Lung[Title])) OR (Non-Small Cell Lung Cancer[Title])) OR (Non-Small-Cell Lung Carcinoma[Title])) OR (Non Small Cell Lung Carcinoma[Title])) OR (Nonsmall Cell Lung Cancer[Title])) OR (Non-Small Cell Lung Carcinoma[Title])) OR (NSCLC[Title])) AND ((((((EGFR-mutant[Title/Abstract]) OR (EGFR-mutated[Title/Abstract])) OR (EGFR-activating mutations[Title/Abstract])) OR (EGFR mutant[Title/Abstract])) OR (Epidermal Growth Factor Receptor Mutant[Title/Abstract])) OR (EGFR-Sensitive Mutations[Title/Abstract]))) AND (((unresectable[Title/Abstract]) OR (inoperable[Title/Abstract])) OR (not resectable[Title/Abstract]))) AND ((((phase III[Title/Abstract]) OR (stage III[Title/Abstract])) OR (locally advanced[Title/Abstract])) OR (localized advanced[Title/Abstract])) |
| Embase |
| ('non small cell lung cancer'/de OR 'nsclc':ti) AND  ('egfr mutation'/de OR ('egfr' NEAR/3 (mutant* OR mutat*)):ti,ab) AND  ('unresectable':ti,ab OR 'inoperable':ti,ab OR 'locally advanced':ti,ab) AND  [article]/lim AND [english]/lim |
| Cochrane Library |
| #1 (NSCLC):ti,ab,kw OR ("non-small cell lung cancer"):ti  #2 ("EGFR mutation"):ti,ab,kw OR ("EGFR-mutant"):ti,ab,kw OR ("EGFR-mutated"):ti,ab,kw  #3 (unresectable):ti,ab,kw OR (inoperable):ti,ab,kw OR ("locally advanced"):ti,ab,kw  #4 #1 AND #2 AND #3 |
| Web of Science databases |
| TS=("non-small cell lung cancer" OR "NSCLC") AND TS=("EGFR-mutant" OR "EGFR mutation" OR "EGFR-mutated" OR "epidermal growth factor receptor mutant") AND TS=("unresectable" OR "inoperable" OR "locally advanced" OR "stage III") |

**Supplementary Table 3**

Node-splitting analysis of inconsistency.

| **Endpoint** | **Comparison** | **Direct effect** | **Indirect effect** | **Network Effect** | **P** |
| --- | --- | --- | --- | --- | --- |
| Progression-free Survival | CRT+Durva vs. CRT+EGFR-TKI  CRT+EGFR-TKI vs. EGFR-TKI | -1.70 (-2.70, -0.72)  0.50 (-0.06, 1.10) | -0.99 (-2.20, 0.04)  0.69 (-0.33, 1.70) | -1.30 (-2.00, -0.66)  0.71 (0.01, 1.40) | 0.310  0.720 |
| Overall Survival | CRT+Durva vs. CRT+EGFR-TKI  CRT+EGFR-TKI vs. EGFR-TKI | -0.20 (-1.10, 0.74)  0.39 (-0.42, 1.20) | -0.27 (-1.40, 0.80)  -0.25 (-1.30, 0.73) | -0.27 (-0.86, 0.33)  0.20 (-0.42, 0.70) | 0.917  0.250 |
| All-grade TRAEs | CRT+Durva vs. CRT  CRT+Durva vs. CRT+EGFR-TKI  CRT vs. CRT+EGFR-TKI | -15.00 (-47.00, -2.50)  0.13 (-2.60, 2.80)  1.90 (0.01, 3.90) | -1.80 (-5.20, 1.50)  -15.00 (-47.00, -0.63)  17.00 (2.60, 50.00) | -2.50 (-6.40, -0.80)  -0.32 (-3.50, 1.40)  2.20 (0.71, 4.50) | 0.041  0.035  0.036 |
| Grade≥3 TRAEs | CRT+Durva vs. CRT  CRT+Durva vs. CRT+EGFR-TKI  CRT vs. CRT+EGFR-TKI | 0.00 (-3.00, 2.80)  -1.40 (-4.20, 1.20)  1.50 (-0.15, 3.50) | -2.90 (-6.30, 0.12)  1.50 (-1.90, 5.00)  -1.40 (-5.40, 2.50) | -1.40 (-3.70, 0.89)  -0.33 (-2.50, 1.90)  1.10 (-0.63, 2.80) | 0.151  0.155  0.150 |
| Radiation Pneumonitis | CRT+Durva vs. CRT  CRT+Durva vs. CRT+EGFR-TKI  CRT vs. CRT+EGFR-TKI | -0.26 (-2.00, 1.40)  -0.43 (-2.20, 1.10)  0.50 (-0.14, 1.20) | -0.93 (-2.80, 0.75)  0.24 (-1.60, 2.00)  -0.20 (-2.60, 2.10) | -0.55 (-1.80, 0.60)  -0.10 (-1.30, 1.00)  0.46 (-0.16, 1.10) | 0.572  0.570  0.574 |

**Supplementary Table 4**

Leave-one-out sensitivity analysis for all-grade treatment-related adverse events (TRAEs).

| Regimen (vs. CRT) | Original Network Effect, OR (95% CrI) | After Exclusion Effect, OR (95% CrI) |
| --- | --- | --- |
| CRT+Durva | 13.00 (2.30, 610.00) | 8.20 (1.40, 130.00) |
| CRT+EGFR-TKI | 8.90 (2.00, 88.00) | 7.70 (2.10, 41.00) |
| EGFR-TKI+RT | 1.60 (0.06, 46.00) | 1.60 (0.11, 29.00) |

**
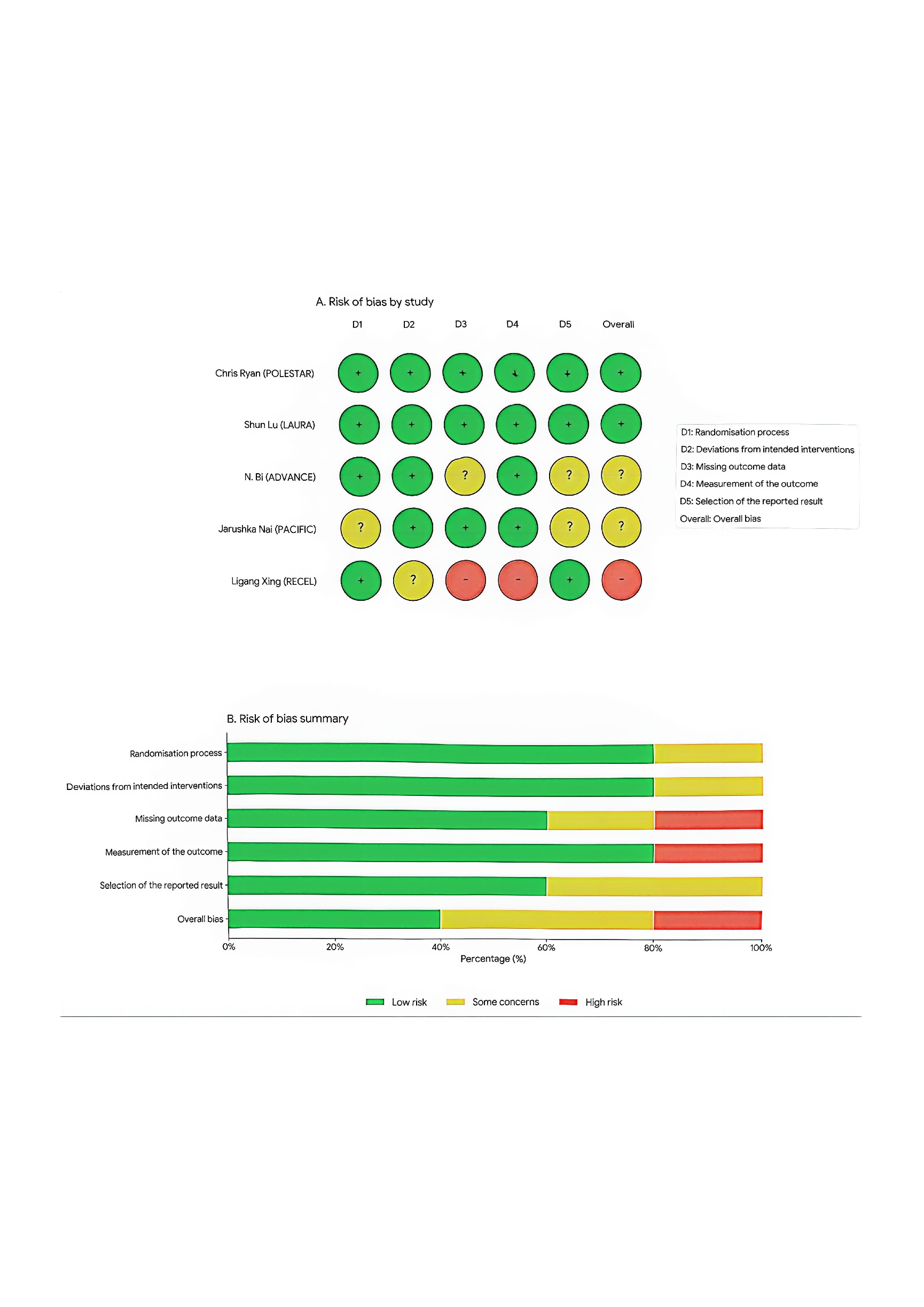
**

**C**

**
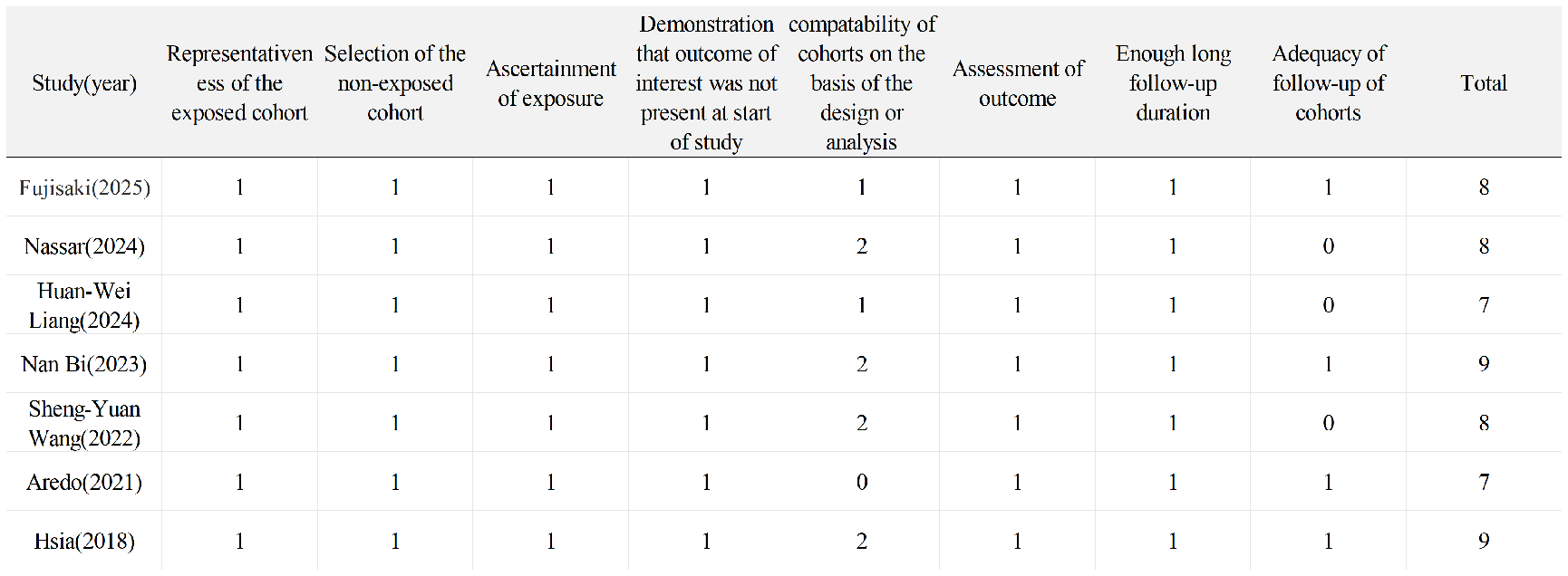
**

**Supplementary Figure 1**

Summary of results from the bias risk assessment of RCTs using the Cochrane risk of bias Tool(A,B). All 7 retrospective cohort studies utilizing the Newcastle-Ottawa Scale(C).

**A**


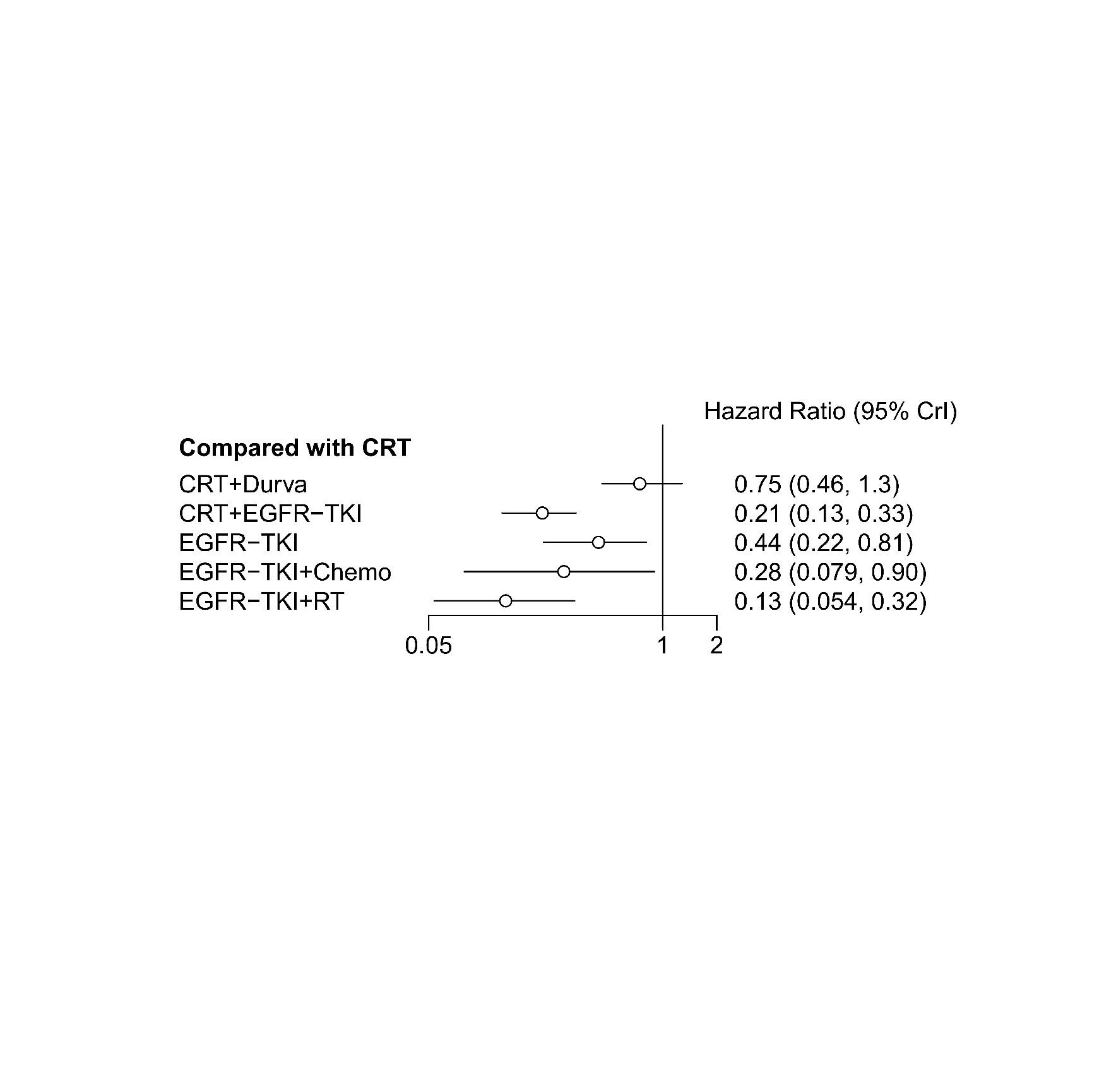


**B**

**
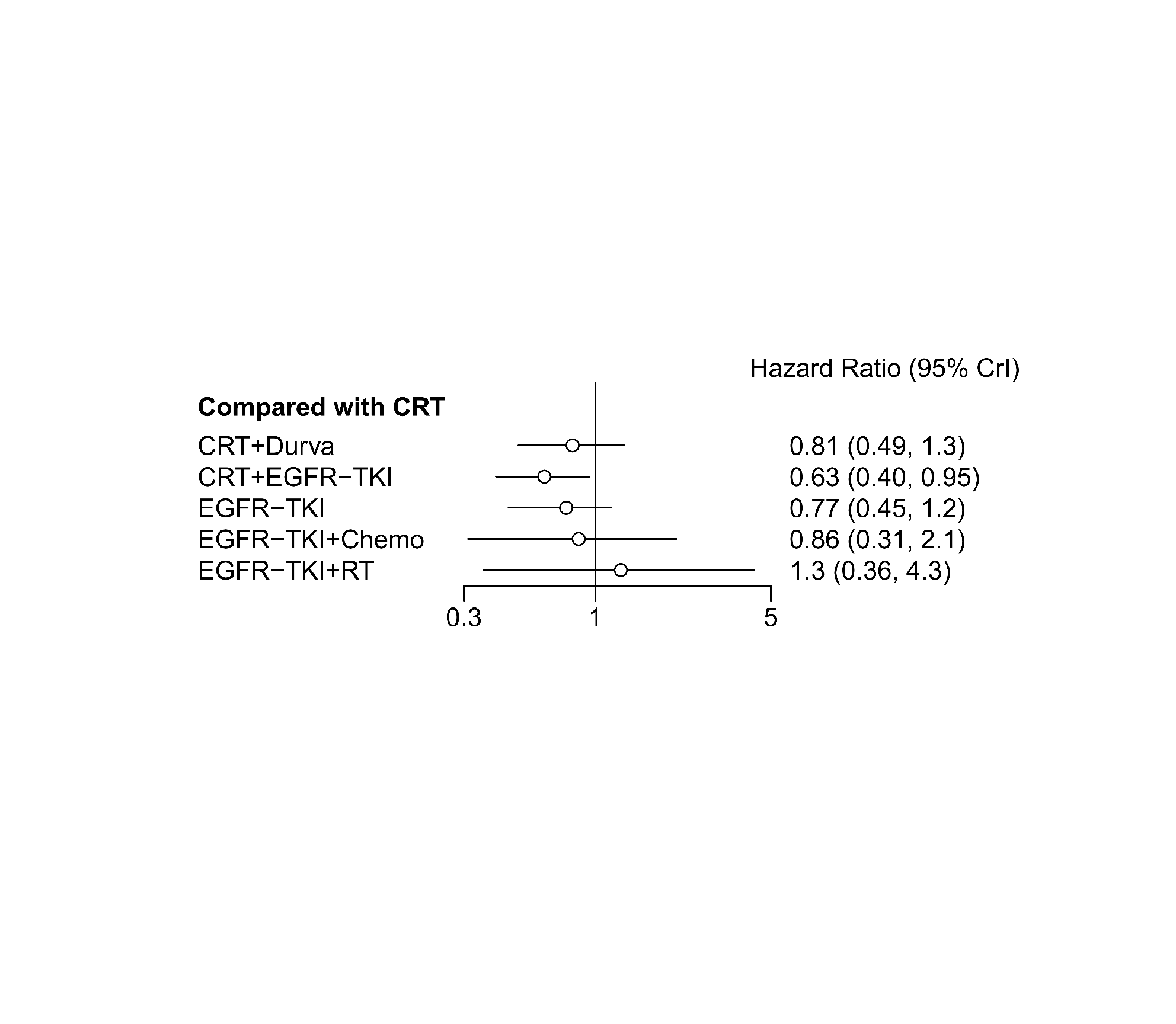
**

**C**

**
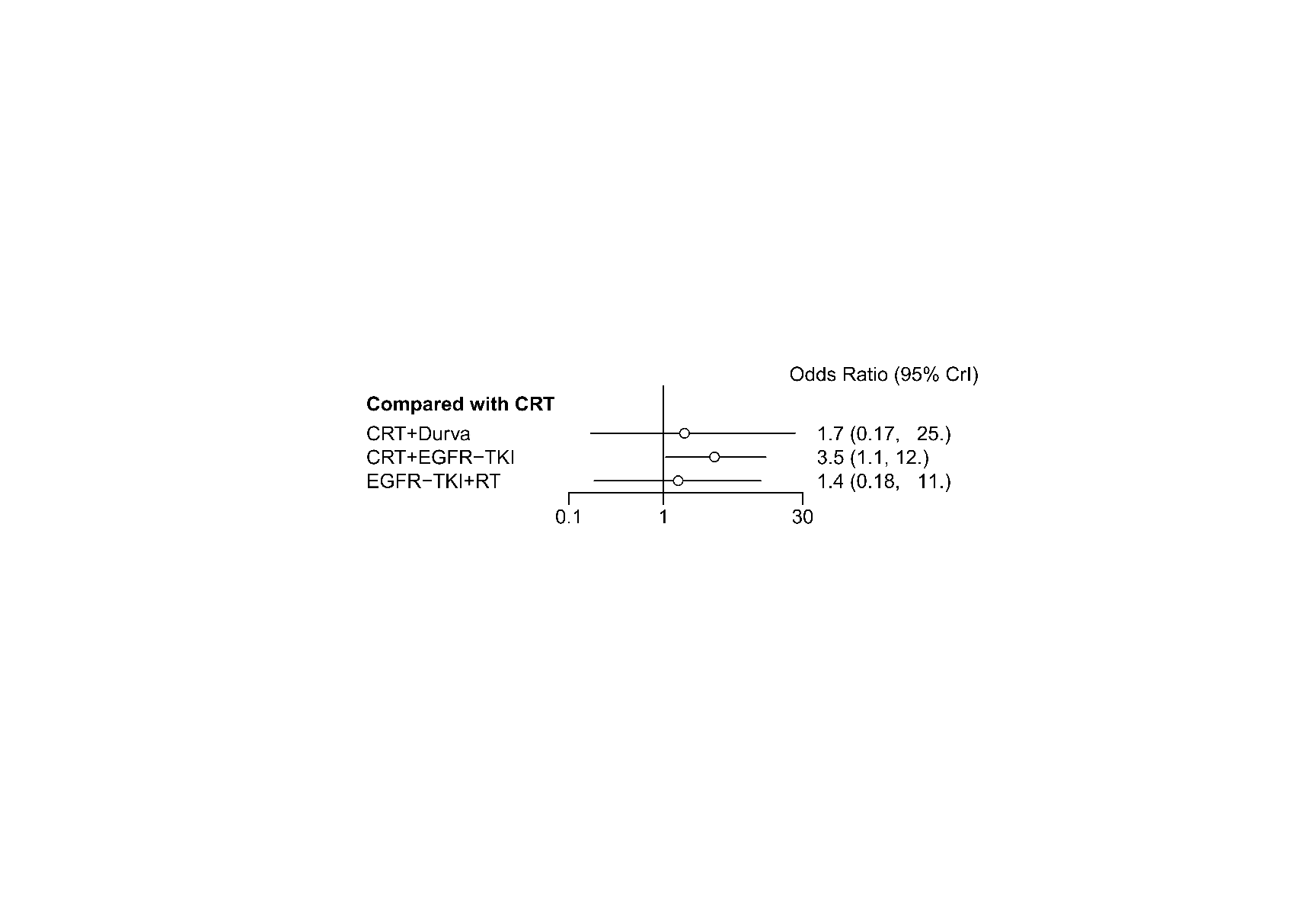
**

**D**

**
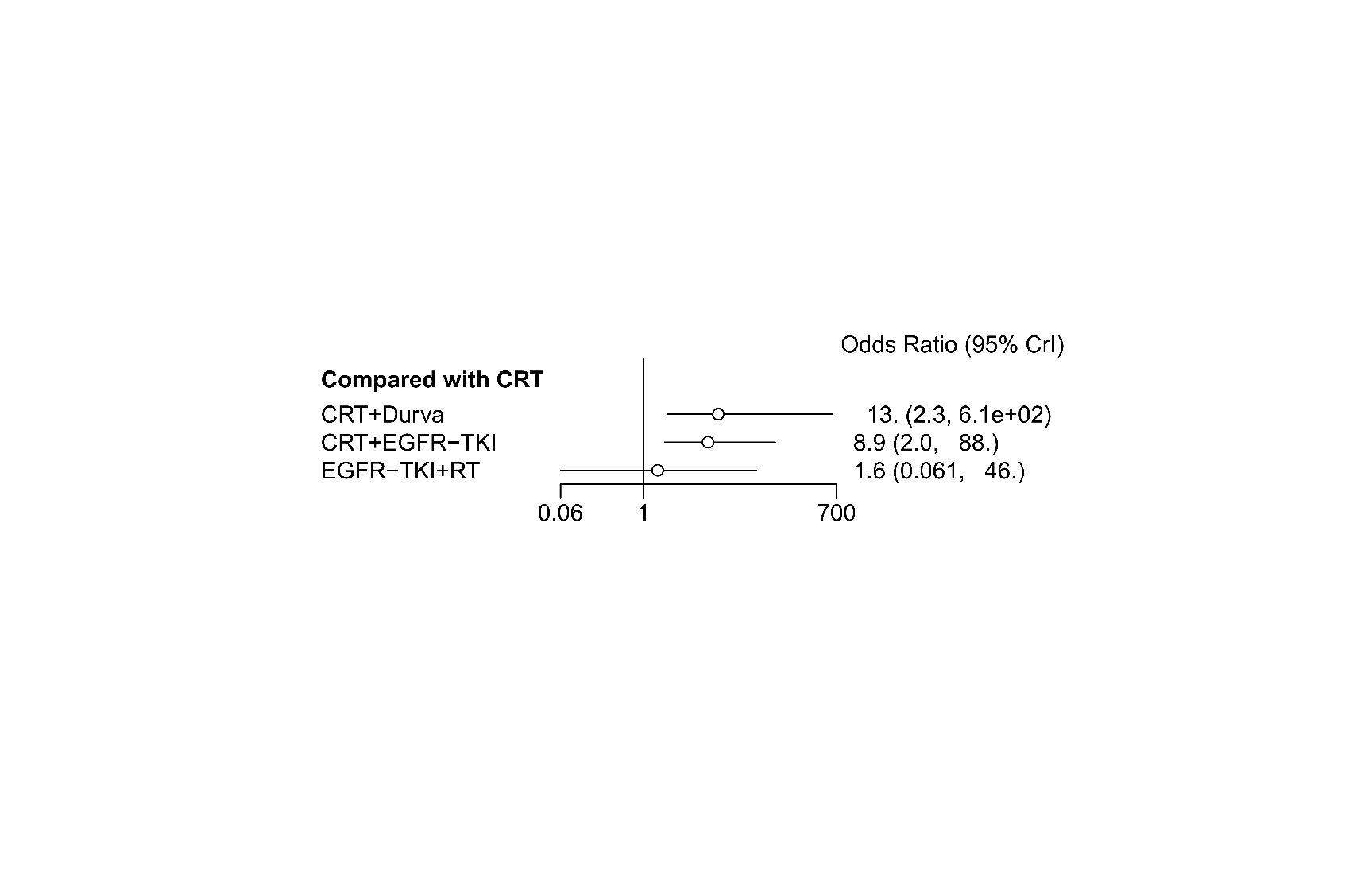
**

**E**

**
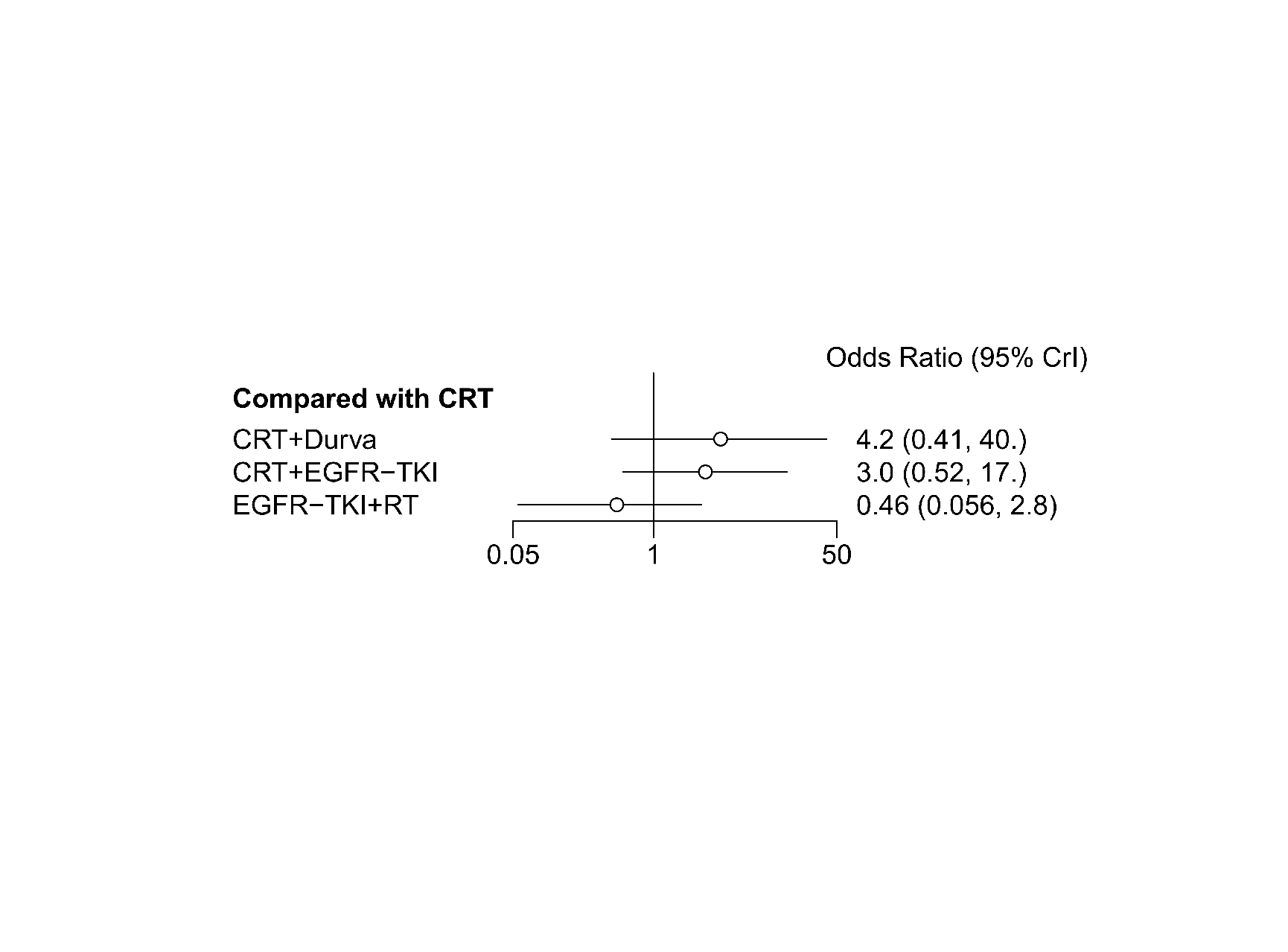
**

**F**

**
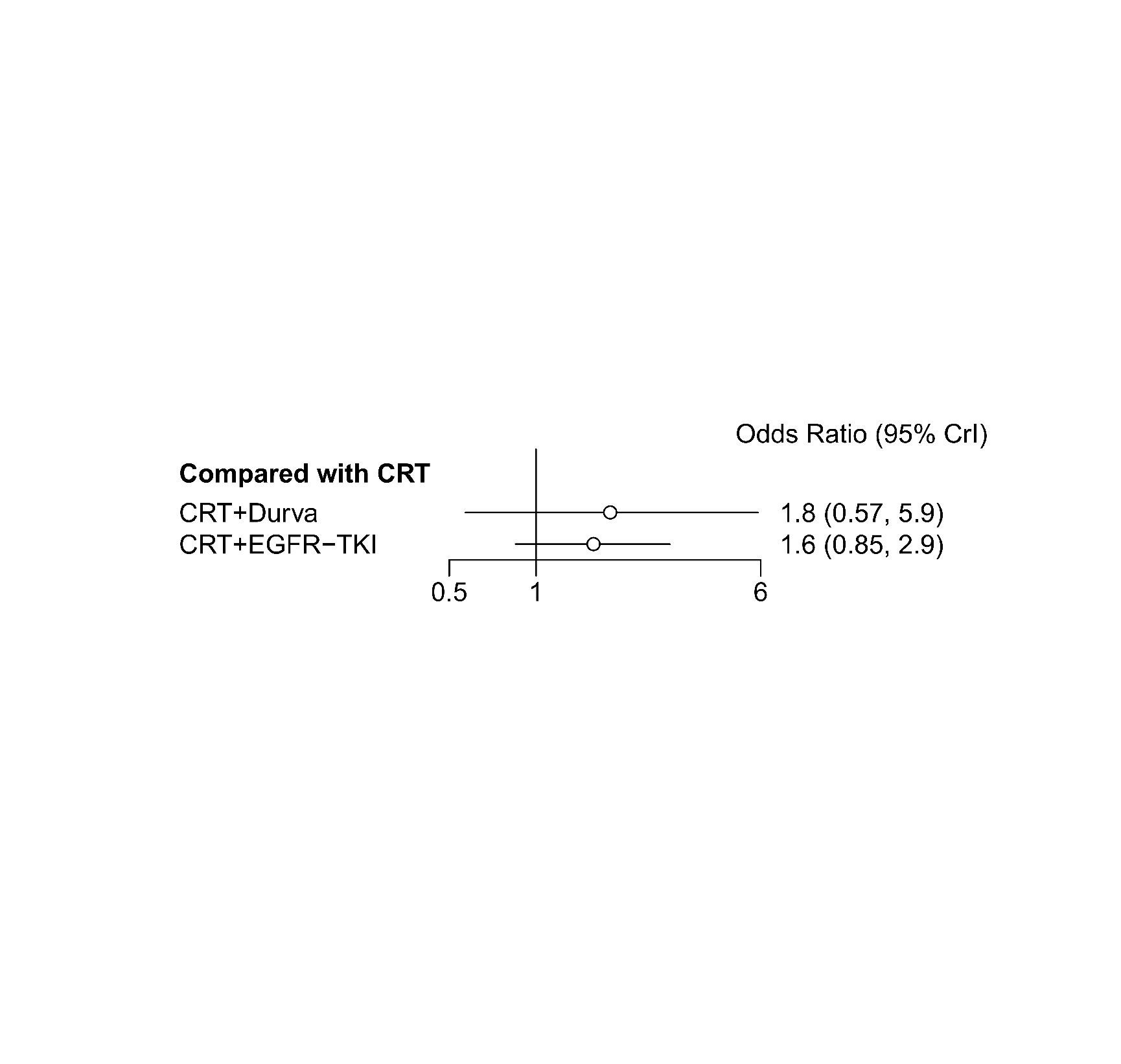
**

**Supplementary Figure 2**

Forest plot of the network meta-analysis.(A) HRs for PFS, (B) HRs for OS,(C) ORs for ORR,(D) ORs for TRAEs,(E) ORs for Grade≥3 TRAEs,(F) ORs for RP. CRT: Chemoradiotherapy, EGFR-TKI: Epidermal growth factor receptor-Tyrosine kinase inhibitor, EGFR-TKI+Chemo: EGFR-TKI in combination with chemotherapy, EGFR-TKI+RT: EGFR-TKI integrated with radiotherapy, CRT+Durva: durvalumab following chemoradiotherapy, HR: hazard ratio, OR: odds ratio, PFS: Progression-free survival, OS: Overall survival, ORR: Objective response rate, TRAEs: Treatment-related adverse events, RP: Radiation pneumonitis.


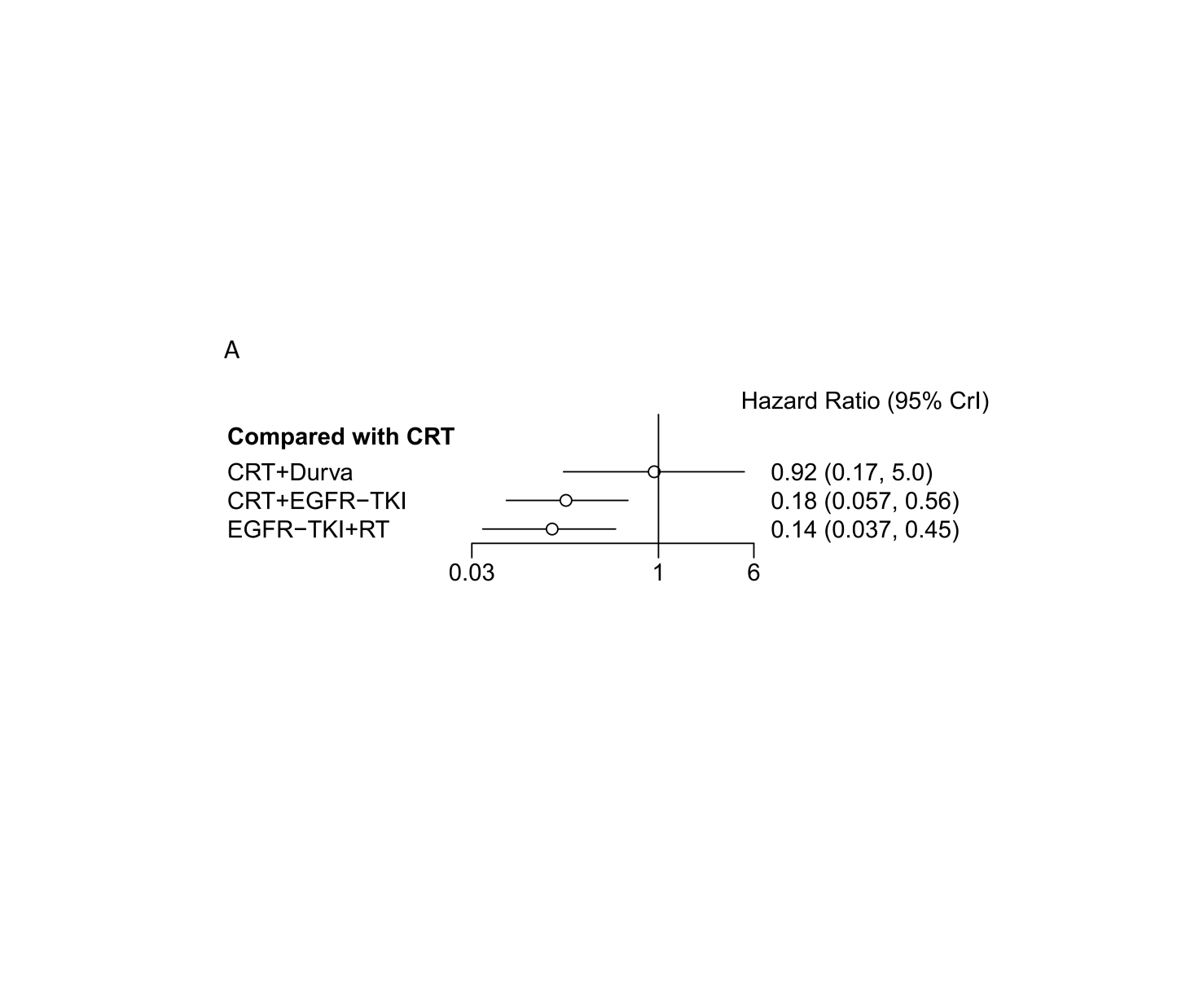


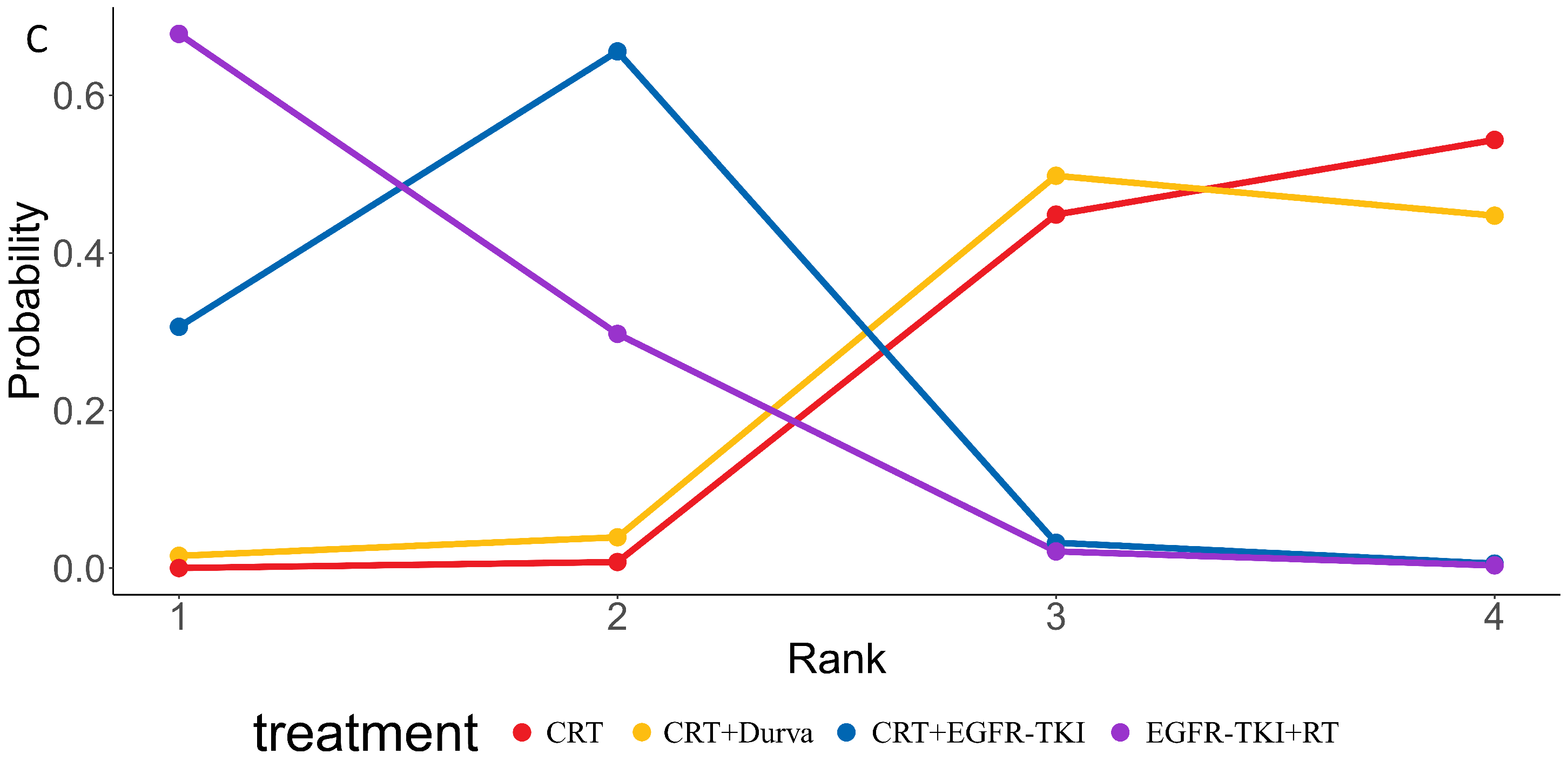

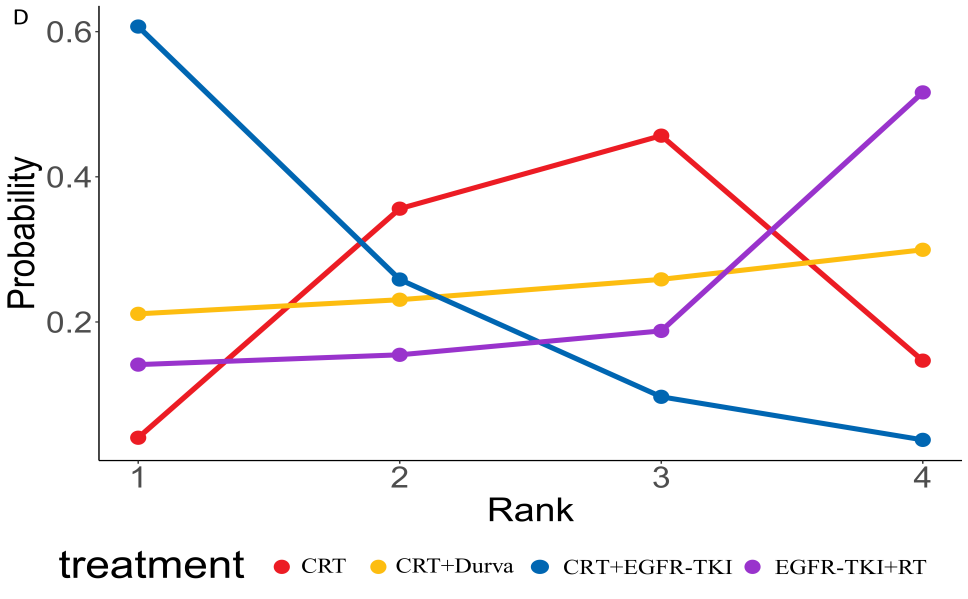


**Supplementary Figure 3**

Forest plots and ranking profiles for RCT-only analysis.(A) HRs for PFS, (B) HRs for OS.The line graph shows the probability of ranking from first to last for each treatment in terms of PFS and OS. (C) PFS. (D) OS. CRT: Chemoradiotherapy, Durva: Durvalumab, EGFR-TKI: Epidermal growth factor receptor-tyrosine kinase inhibitor, RT: radiotherapy.


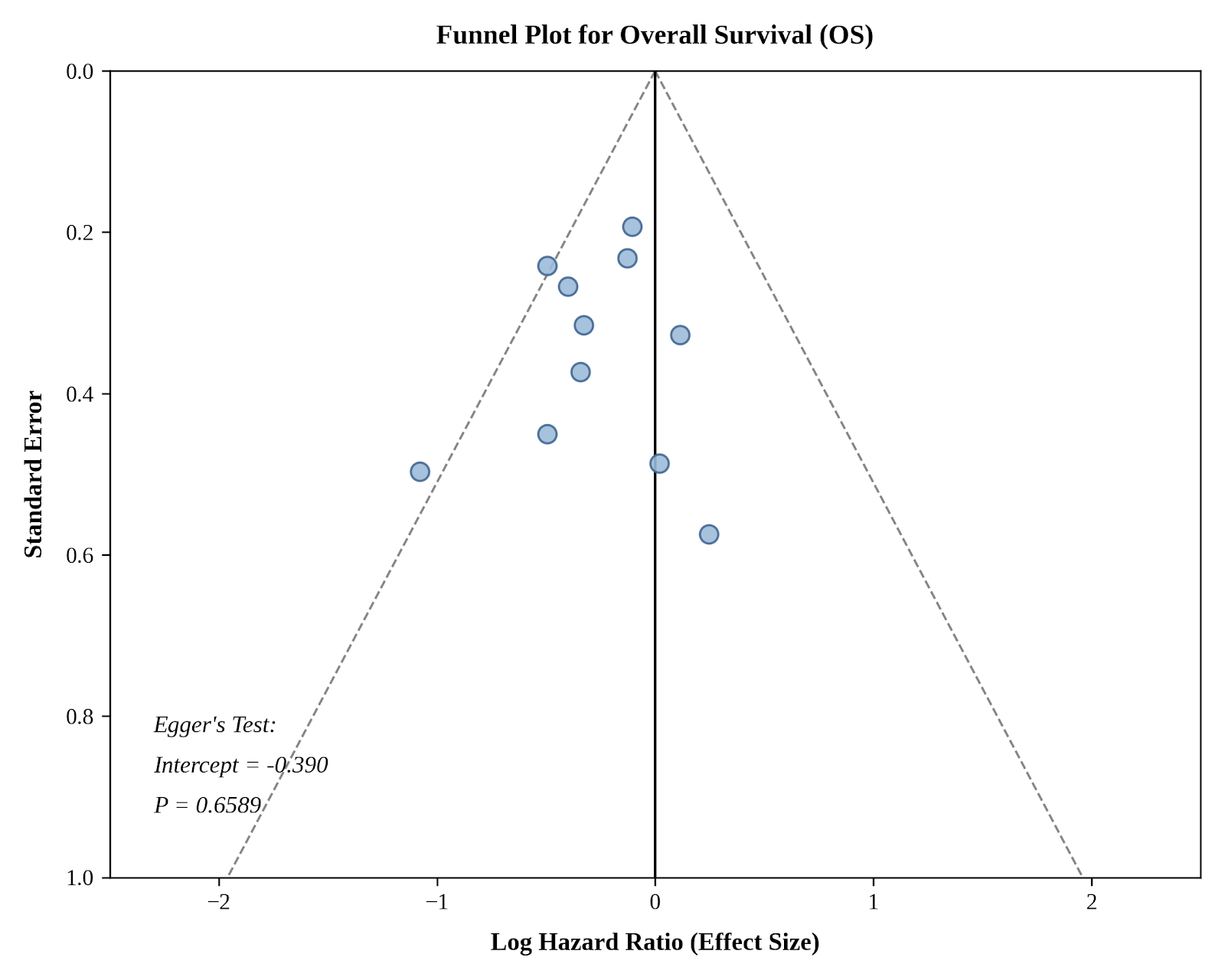

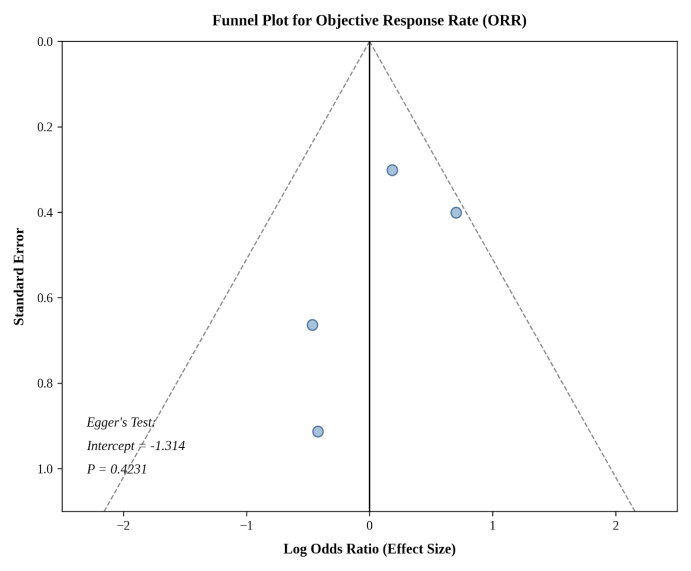

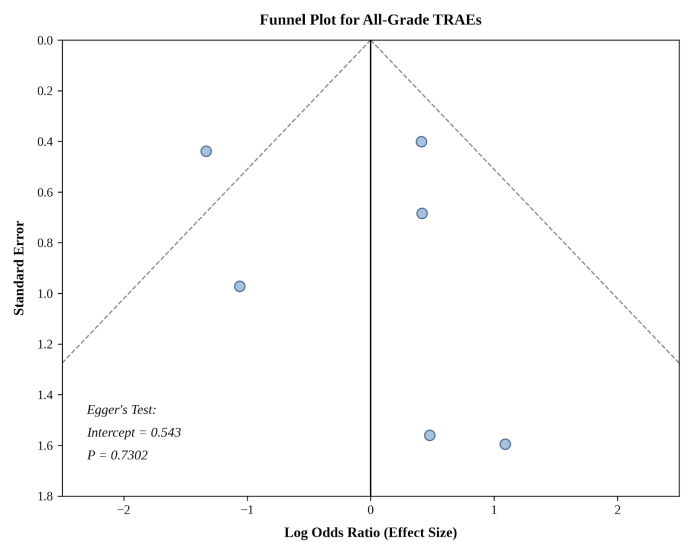

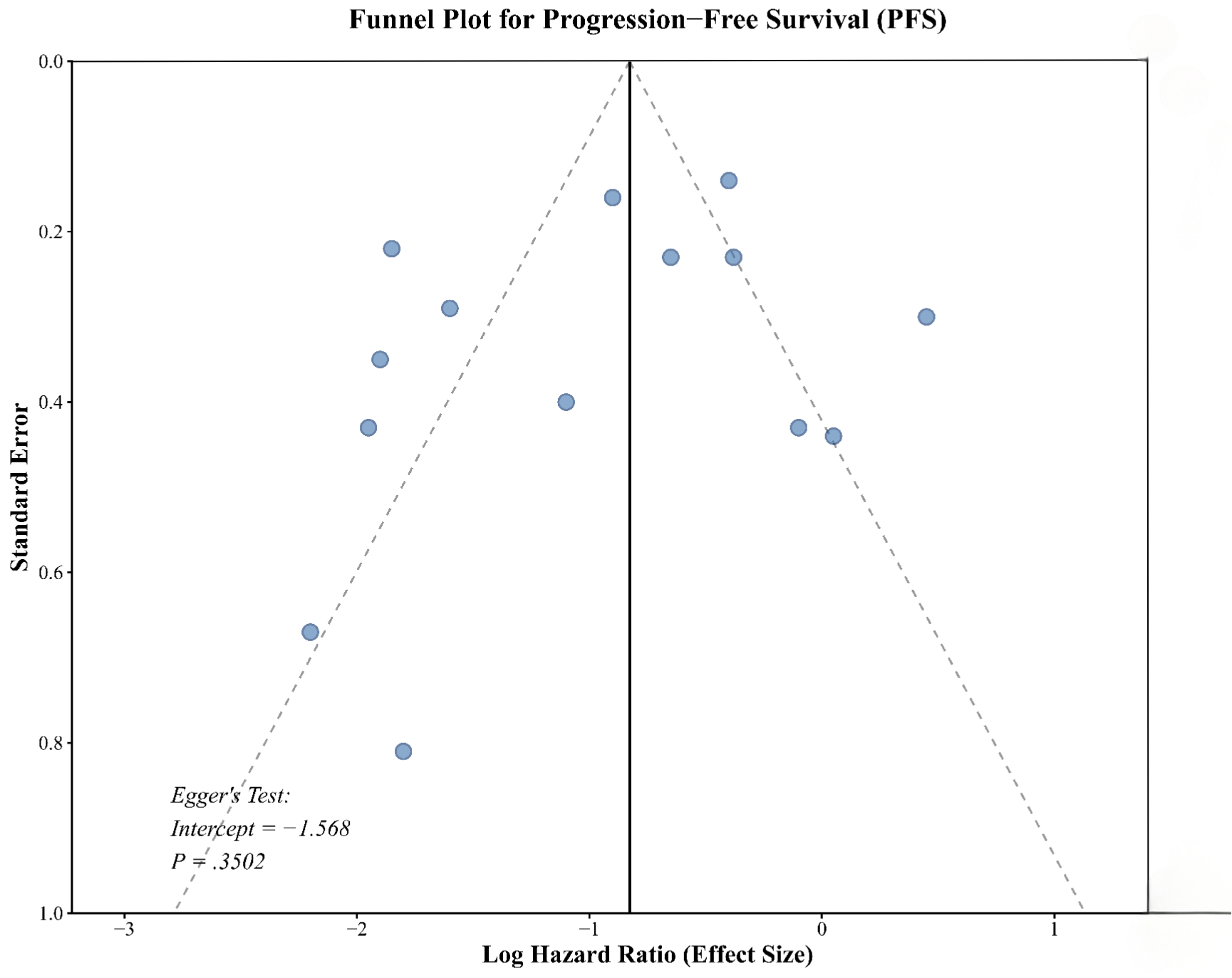

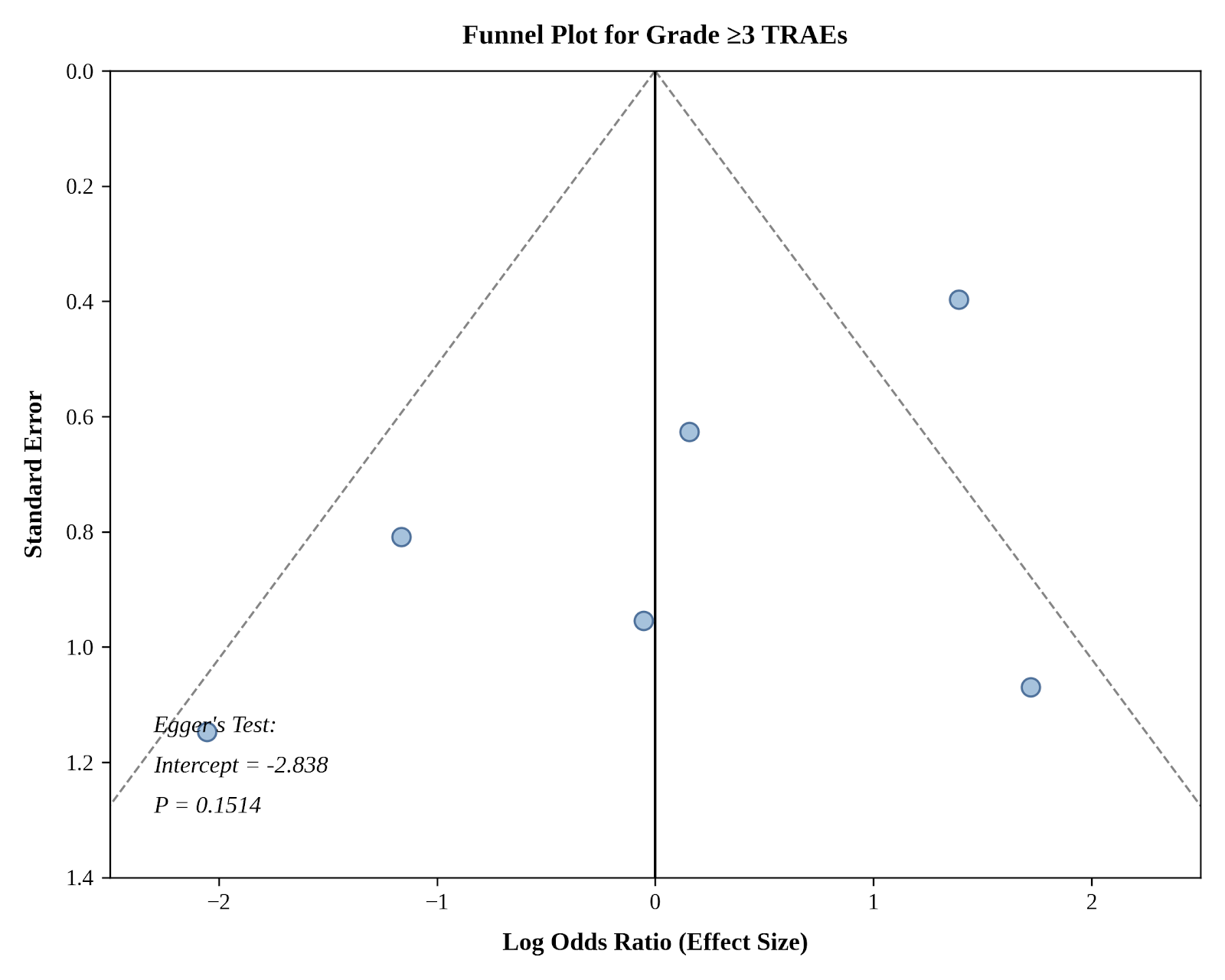

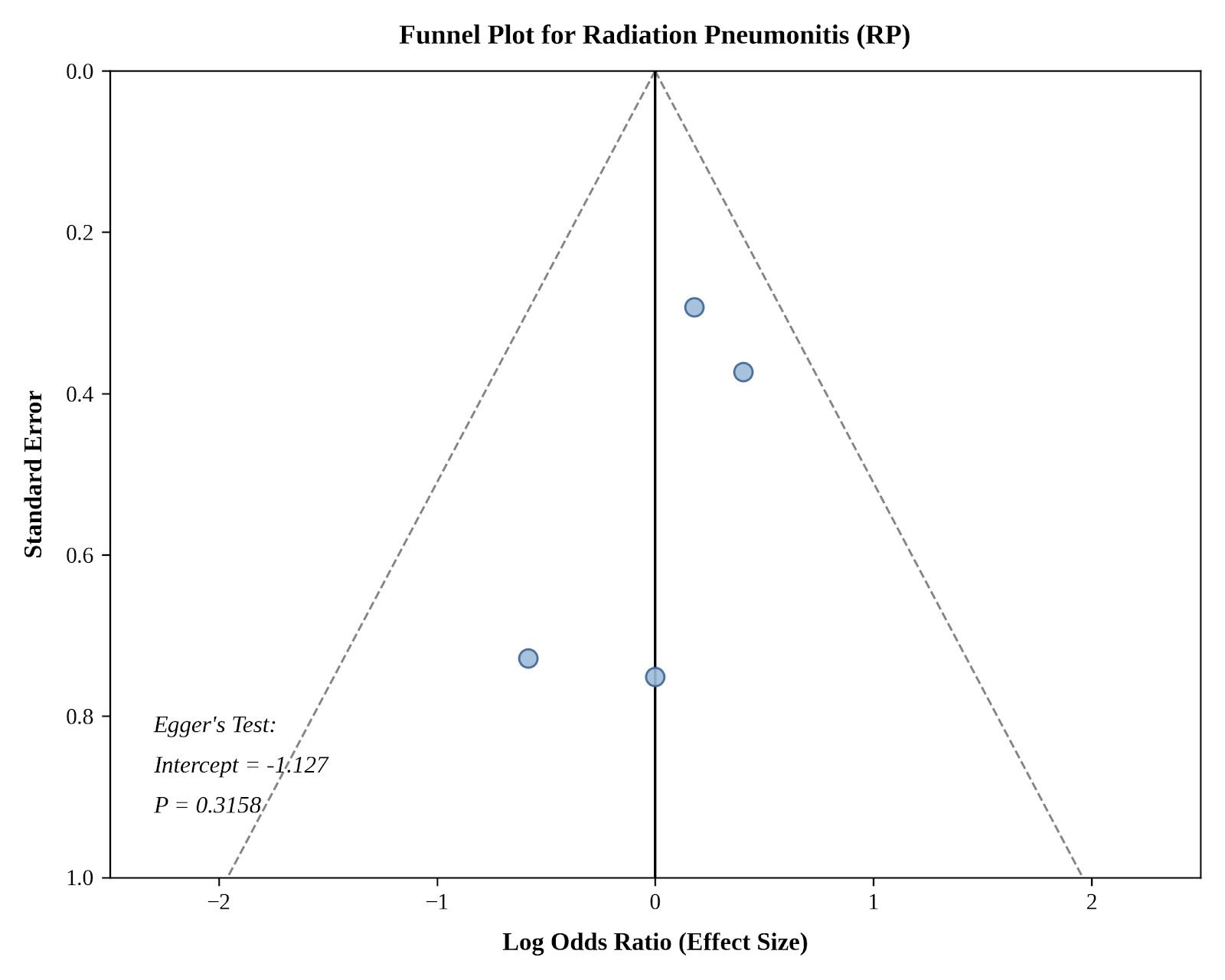


**Supplementary Figure 4**

Comparison-adjusted funnel plot for assessing publication bias in progression-free survival (PFS), overall survival (OS), objective response rate (ORR), all-grade treatment-related adverse events (TRAEs), grade ≥ 3 TRAEs, and radiation pneumonitis (RP). Each blue circle represents an individual study included in the network meta-analysis. The horizontal axis represents the log hazard ratio (effect size) , while the vertical axis represents the standard error. The dashed lines indicate the 95% confidence intervals. The visual distribution of studies is relatively symmetrical within the funnel, suggesting no obvious evidence of asymmetry. Since P > 0.05, there is no statistically significant evidence of publication bias for all outcome in this study.
